# Automated Seed Morphometry Identifies Morphotypes Associated with Major Grapevine Haplotypic Lineages

**DOI:** 10.3390/plants15182774

**Published:** 2026-09-10

**Authors:** Emilio Cervantes, José Javier Martín-Gómez, Ángel Anocibar Beloqui, Félix Cabello Sáenz de Santa María, Gregorio Muñoz Organero, Jorge Cunha, Ángel Tocino, José Luis Rodríguez-Lorenzo

**Affiliations:** 1 Instituto de Recursos Naturales y Agrobiología, Consejo Superior de Investigaciones Científicas, Cordel de Merinas 40, 37008 Salamanca, Spain; jjavier.martin@irnasa.csic.es; 2 Abadía Retuerta, Sardón de Duero, 47340 Valladolid, Spain; 3 Instituto Madrileño de Investigación y Desarrollo Rural Agrario y Alimentario (IMIDRA), Finca El Encín, 28805 Alcalá de Henares, Spain; 4 Instituto Nacional de Investigação Agrária e Veterinária, Quinta da Almoinha, 2565-191 Dois Portos, Portugal; jorge.cunha@iniav.pt; 5 Departamento de Matemáticas, Facultad de Ciencias, Universidad de Salamanca, Plaza de la Merced 1-4, 37008 Salamanca, Spain; 6 Plant Developmental Genetics, Institute of Biophysics v.v.i, Academy of Sciences of the Czech Republic, Královopolská 135, 612 65 Brno, Czech Republic; rodriguez@ibp.cz

**Keywords:** computational phenomics, curvature analysis, elliptic Fourier analysis (EFA), geometric morphometrics, grapevine, *J* index, seed morphometrics, seed morphotypes, *Vitis vinifera* L.

## Abstract

High-throughput DNA sequencing has revealed that the extensive diversity of *Vitis vinifera* L. cultivars is structured into several major genetic lineages. Identifying phenotypic traits that reflect these genetic relationships remains an important challenge for grapevine taxonomy, germplasm characterization, and archaeobotany. Here, we present an automated computational workflow implemented in R that reconstructs seed outlines from Elliptic Fourier Descriptors (EFDs) and calculates the *J* index, a measure of similarity to predefined reference morphotypes, together with curvature profiles along the seed outline. The workflow is reproducible and suitable for high-throughput morphometric analysis. A dataset comprising 2895 seeds from 143 populations representing 85 *V. vinifera* cultivars was initially organized into three groups according to available pedigree information and previously characterized seed morphotypes. Geometric reference models representing the Hebén, Savagnin Blanc, and Muscat morphotypes were then used to calculate *J* index values for each population. Based on these values, 74 populations were assigned unambiguously to one of the three reference morphotypes, whereas 69 showed similar affinities to two or three models and were therefore classified as intermediate or undifferentiated. The three primary morphometric groups comprised 41 populations assigned to the Hebén morphotype, 12 to the Savagnin Blanc morphotype, and 21 to the Muscat morphotype. Curvature analysis of the reconstructed seed outlines was subsequently used to characterize local geometric variation and to identify representative populations within each group. The resulting morphotypes showed substantial correspondence with major grapevine haplotypic lineages while also revealing intermediate phenotypes associated with the complex genetic and developmental history of cultivated grapevine. Overall, the workflow provides an objective and reproducible approach for high-throughput seed phenotyping and represents a complementary phenotypic tool for investigating grapevine genetic diversity, germplasm characterization, and the relationships between seed morphology and genetic lineage.

## 1. Introduction

The advent of high-throughput DNA sequencing has transformed plant biology by providing unprecedented insights into the genetic architecture, population structure, and evolutionary history of cultivated species [1,2,3,4]. In grapevine (*Vitis vinifera* L.), extensive genotyping, whole-genome sequencing, and haplotype-resolved analyses have enabled the reconstruction of complex pedigrees [5,6,7,8,9,10], the characterization of clonal diversity [11,12], and the identification of genomic regions associated with key agronomic traits [13,14,15,16,17,18,19]. However, the rapid expansion of genomic information has also highlighted a persistent phenomics bottleneck. Although genetic variation can now be characterized at genome-wide resolution, the acquisition of standardized and comparable phenotypic data remains constrained by labour-intensive measurements, heterogeneous methodologies, and descriptors that do not always capture the complexity of biological form. Consequently, integrating genomic and phenotypic datasets remains a major challenge.

Grapevine phenotyping traditionally relies on multi-organ ampelography, including the descriptor system developed by the International Organisation of Vine and Wine (OIV) descriptors [20]. Although vegetative and cluster traits provide important criteria for cultivar identification, many of these characters exhibit substantial phenotypic plasticity. In contrast, the lignified seed endocarp undergoes sclerification during maturation and provides a relatively rigid structure that is less affected by seasonal and canopy-management conditions [21,22,23]. Consequently, seed shape can preserve morphological signals associated with genetic relationships, geographic differentiation, and the historical processes of grapevine domestication. Morphometrics, defined as the quantitative analysis of biological form, therefore provides an effective approach for bridging genotype and phenotype. In *V. vinifera*, seed morphology is particularly informative because it can preserve evolutionary and taxonomic signals that may be less apparent in highly plastic vegetative organs.

The taxonomic value of grapevine seeds has long been recognized in ampelography and archaeobotany [24,25,26]. More recently, geometric morphometric analyses have identified characteristic seed morphotypes associated with major grapevine genetic lineages [27], including the Hebén lineage [23], an important founder of the Iberian grapevine gene pool, the Savagnin Blanc lineage, an important component of Central European viticulture, and the Muscat lineage, which represents a distinct morphogenetic group [28,29,30,31]. The recurrence of these morphotypes suggests that seed morphology contains biologically meaningful variation related to genetic relationships and the historical diversification of grapevine.

Quantifying these relationships requires objective, reproducible, and scalable analytical methods. Elliptic Fourier Analysis (EFA) has become a powerful approach for the quantitative characterization of grapevine seed morphology because it provides a comprehensive mathematical description of closed two-dimensional outlines [32,33,34]. Unlike conventional linear measurements, such as seed length, width, or their ratios, which capture only a limited number of geometric properties, EFA represents the entire contour through a series of harmonic functions. The boundary coordinates are expressed as parametric functions of the position along the contour and decomposed into trigonometric components, or harmonics, that describe progressively finer aspects of shape. Each harmonic is defined by four Fourier coefficients (A_n_, B_n_, C_n_, and D_n_), which together describe the geometry of the outline. Lower-order harmonics capture broad features such as overall shape, length, and width, whereas higher-order harmonics describe increasingly localized characteristics, including apex sharpness, notch depth, and basal curvature. EFA therefore provides a quantitative and reproducible representation of seed outlines that can be used for subsequent geometric analyses.

Following normalization, Elliptic Fourier Descriptors (EFDs) provide standardized representations of seed shape that can be compared among samples independently of differences in size, orientation, and position [29,31]. Increasing the number of harmonics progressively improves the approximation of the original biological contour. The resulting reconstructed outlines can therefore serve as a common geometric basis for additional quantitative descriptors. Among these, the *J* index provides a measure of similarity between a biological outline and a predefined reference morphotype, whereas curvature analysis quantifies local geometric variation along the contour. The combination of these complementary descriptors makes it possible to characterize seed morphology at both global and local scales.

Curvature measures the rate of directional change along a contour, allowing the precise identification of local maxima, minima, inflection points, and other anatomically relevant features. Algorithms for estimating curvature from sequences of points defining closed planar outlines were originally developed to quantify root apex curvature in wild-type and ethylene-mutant *Arabidopsis* seedlings [35] and were subsequently adapted for the quantitative analysis of characteristic points along grapevine seed outlines [28,29,30,31]. Together with the *J* index, curvature analysis provides complementary information by combining global morphotype similarity with local geometric variation, thereby enabling a detailed mathematical characterization of seed architecture. Despite these advances, no fully automated workflow is currently available that reconstructs seed outlines from Elliptic Fourier Descriptors while simultaneously calculating *J* index values and curvature profiles within a reproducible, high-throughput analytical framework.

Seed morphology is a complex quantitative trait. Polygenic inheritance, developmental processes during seed formation, environmental variation, and complex multi-parental backgrounds can produce intermediate phenotypes that are difficult to classify using single morphological characters. A combined approach based on global outline similarity and local curvature may therefore provide greater discriminatory power than conventional visual assessment or simple linear measurements. Such an approach is particularly relevant for large germplasm collections, where manual evaluation of thousands of seed outlines is impractical.

In this study, we present an automated computational workflow implemented in R for the quantitative characterization of grapevine seed morphology. Starting from Elliptic Fourier Descriptors, the workflow reconstructs seed outlines and calculates two complementary morphometric descriptors: the *J* index, which quantifies similarity to predefined reference morphotypes, and curvature profiles, which characterize local geometric variation along the seed boundary. We applied the workflow to a dataset comprising 2895 seeds from 143 populations representing 85 *V. vinifera* cultivars. The populations were initially organized according to available pedigree and haplotype information, and their seed outlines were subsequently evaluated against reference morphotypes representing the Hebén, Savagnin Blanc, and Muscat lineages. Populations were then classified according to their *J* index similarity to these reference models, and representative populations from the resulting morphometric groups were selected for detailed curvature analysis. The objective was to develop a reproducible, high-throughput phenotyping framework for characterizing grapevine seed morphotypes and to evaluate the extent to which seed morphology is associated with major grapevine haplotypic lineages.

## 2. Results

### 2.1. Reference Models Used in the Present Study

**Figure 1:**
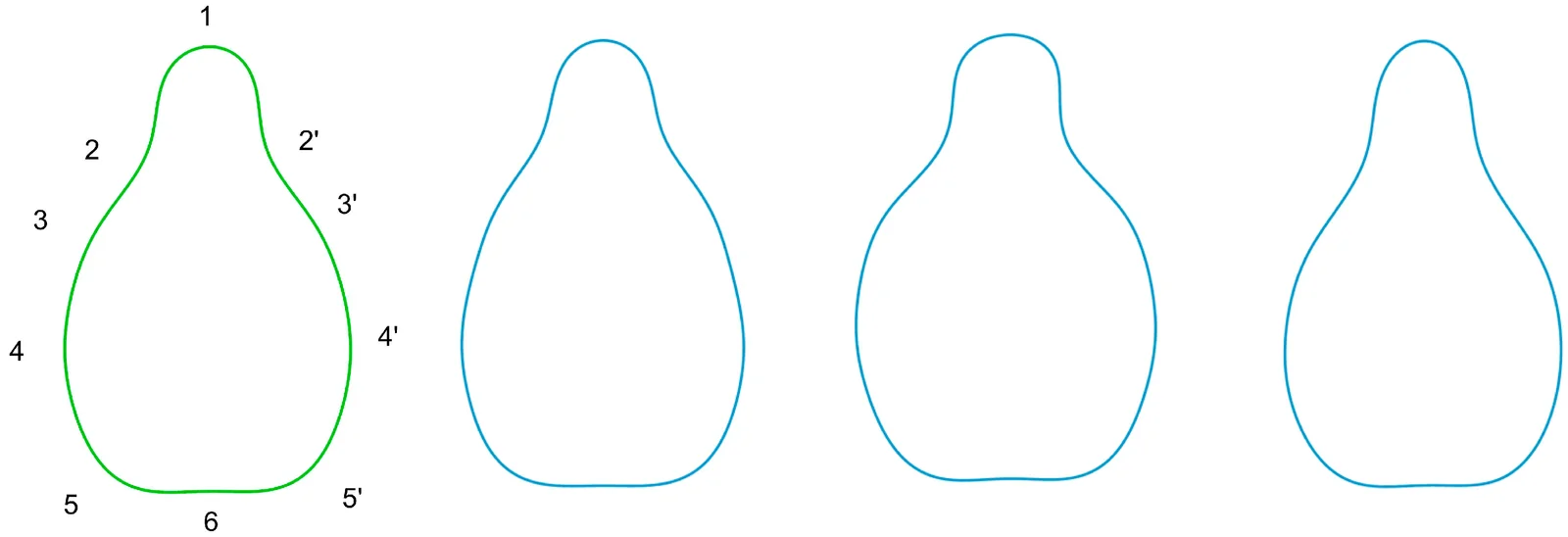
Reference seed morphotypes and curvature landmarks used in this study. **Left**, green: Idealized *Vitis vinifera* seed outline showing the curvature landmarks analyzed. Point 1 indicates the position of maximum positive curvature at the apex of the seed beak; points 2 and 2′ correspond to the curvature minima; points 3 and 3′ mark the transition between the curvature minima and the region of maximum chalaza breadth; points 4 and 4′ indicate the positions of maximum seed width; and points 5 and 5′ correspond to the limits of the basal region. **Right**, blue: Geometric models representing the three reference seed morphotypes (Hebén, Savagnin Blanc, and Muscat) used for *J* index calculations.

Three reference seed morphotypes were used in this study: Hebén, Savagnin Blanc, and Muscat (Figure 1). The Hebén reference model was identical to that employed in previous studies [28,29,30,31] and was generated from the average seed outlines of the Hebén 2020, 2024, and 2025 populations cultivated at IMIDRA. The Savagnin Blanc model was constructed from the average outlines of the cultivars Gewürztraminer and Alfrocheiro (IMIDRA 2020), whereas the Muscat model was generated from the average outlines of Königin der Weingärten and Muscat Hamburg (IMIDRA 2020). The name “Savagnin Blanc” was chosen as the primary reference model because of its key role as an ancestral progenitor of a broad network of Western and Central European cultivars, including Chenin Blanc, Sauvignon Blanc, and Alfrocheiro [36]. Moreover, the curvature profile of the seed outline characteristic of this lineage is highly conserved and, in several progeny, is expressed more clearly than in Savagnin Blanc itself. These three models were selected because they represent the main seed morphotypes identified in our *Vitis vinifera* seed collection. The Hebén morphotype exhibits the highest solidity, the Savagnin Blanc morphotype is characterized by the most negative curvature at the shoulder region, and the Muscat morphotype displays intermediate curvature values. Other characteristics include:

Group I (Hebén lineage): Defined by the lowest average curvature. It features sloped, gently rounded shoulders with shallow lateral indentations, creating a smooth and gradual transition along the upper body toward a moderately pointed beak. Overall, its shape is reminiscent of a Burgundy bottle silhouette.

Group II (Savagnin Blanc lineage): Characterized by strongly negative curvature at the transition from the shoulders toward the beak. Deep lateral indentations produce a pronounced structural constriction above the shoulders before the outline tapers gradually into a broad beak. The overall shape is reminiscent of a high-shouldered Bordeaux bottle.

Group III (Muscat lineage): Characterized by an elongated contour (high aspect ratio) with relatively high curvature and a sharply pointed beak. The lateral shoulders are significantly smoother and less indented than those of Group II.

Figure 1 shows the three reference morphotypes together with an idealized seed outline indicating the curvature landmarks analyzed in this study. Point 1 indicates the position of maximum positive curvature at the apex of the seed beak. Points 2 and 2′ correspond to the minima of curvature within the chalaza–beak notch. Points 3 and 3′ mark the transition between the curvature minima and the region of maximum chalaza breadth. Points 4 and 4′ indicate the positions of maximum seed width, whereas points 5 and 5′ correspond to the limits of the basal region.

### 2.2. Screening of the Seed Collection Using J Index Values

The 143 populations were initially assigned to three groups based on published pedigree information [5,6,8,9,10], following the compilation of Röckel et al. [36]. This preliminary classification is presented in Supplementary Table S1 (Sheets 1 and 2). Subsequently, the automated *J* index analysis was applied to all populations, and the resulting morphometric classification was used to reassign populations according to their similarity to the three reference morphotypes (Table 1; Supplementary Table S1, Sheet 3). Following this classification, the reference morphotypes were designated as Group I (Hebén), Group II (Savagnin Blanc), and Group III (Muscat).

Of the 143 populations analyzed, 74 were assigned unambiguously to one of the three reference morphotypes, whereas the remaining 69 could not be assigned to a single group because they exhibited similar *J* index values for two or more reference models. Of these, 55 showed comparable similarity to the Hebén and Savagnin Blanc morphotypes, four to the Hebén and Muscat morphotypes, and ten exhibited similar *J* index values for all three reference morphotypes.

The final *J* index-based classification comprised 41 populations in Group I (Hebén), 12 in Group II (Savagnin Blanc), and 21 in Group III (Muscat). These populations corresponded to those exhibiting the highest *J* index values relative to their respective reference morphotypes. Seed outlines corresponding to both the preliminary pedigree-based classification and the final *J* index-based classification are provided in the Supplementary Materials.

**Figure 2:**
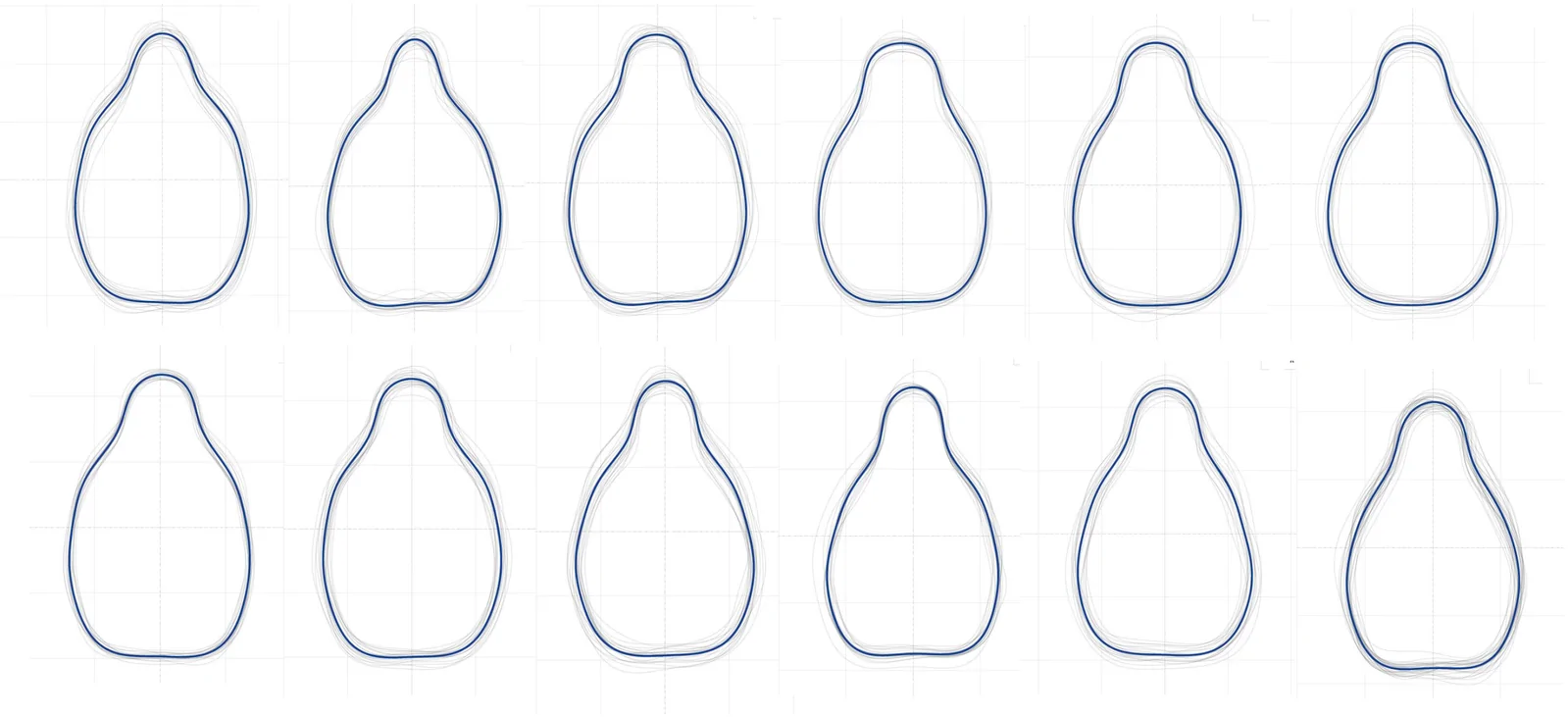
Average seed outlines of populations assigned to Group I (Hebén) following *J* index-based classification. **Top** row (left to right): Alarije IMIDRA 2020, Aledo IMIDRA 2020, Blanquiliña IMIDRA 2020, Cadrete IMIDRA 2020, Esperó de Gall IMIDRA 2020, and Forcallat IMIDRA 2020. **Bottom** row (left to right): Hebén IMIDRA 2020, Hebén IMIDRA 2025, Italia IMIDRA 2020, Italia Valbusenda, Italia Moscatel Romano Valbusenda, and Lariao INIAV Pt. Blue lines indicate the average outline of each population, whereas grey lines represent the individual seed outlines.

Most of the 41 populations assigned to Group I corresponded to cultivars belonging to the Hebén lineage (Table 2; Supplementary Table S1, Sheet 3). Only seven populations had been assigned initially to other lineage groups: two to the Savagnin Blanc group (Blanquiliña and Sauvignon Blanc) and five to the Muscat group, comprising three populations of Italia and two populations of Muscat à Petits Grains Blancs. Representative seed outlines of Group I are shown in Figure 2, whereas the complete set of seed outlines and their corresponding Elliptic Fourier Descriptors (EFDs) is provided in the Supplementary Materials.

**Figure 3:**
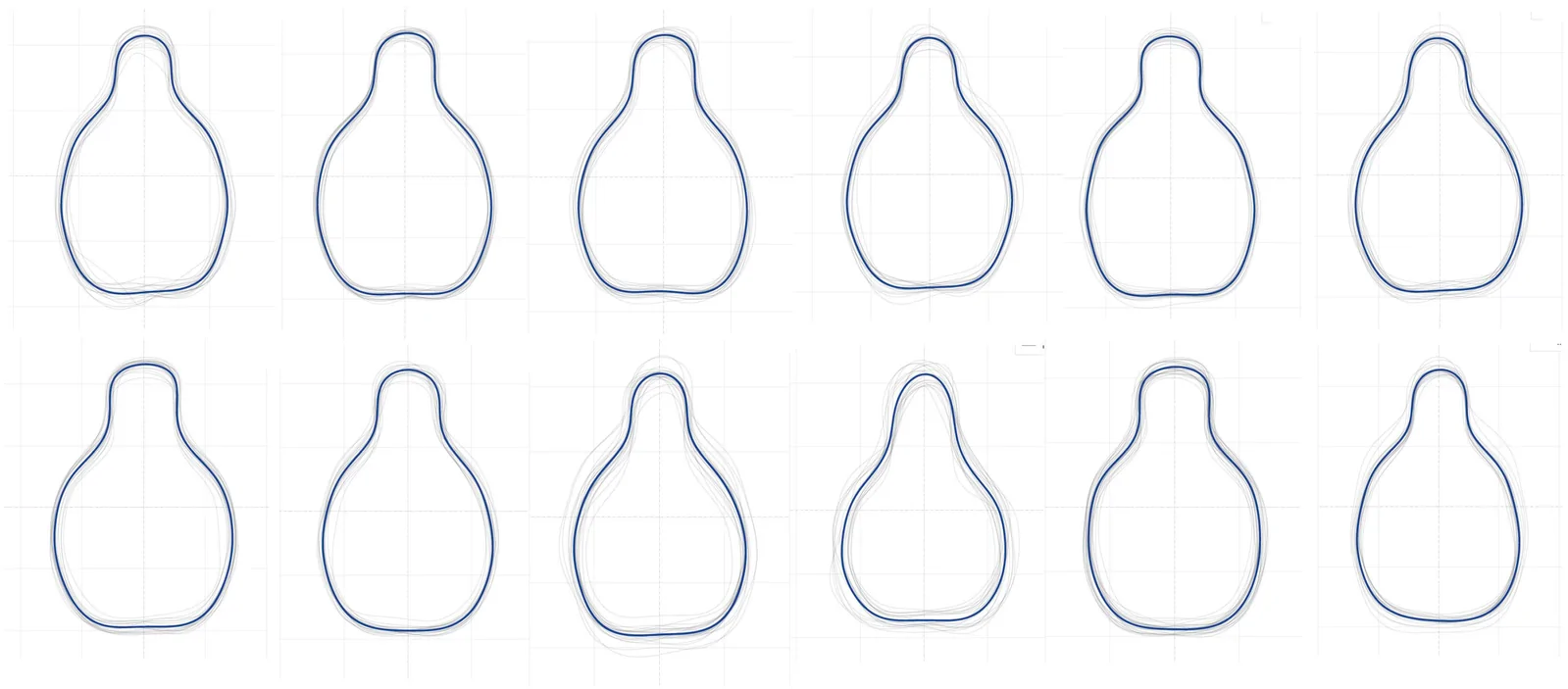
Average seed outlines of populations assigned to Group II (Savagnin Blanc) following *J* index-based classification. **Top** row (left to right): Albarín Blanco IMIDRA 2020, Alfrocheiro IMIDRA 2020, Alfrocheiro INIAV Pt, Beba IMIDRA 2020, Chenin Blanc Valbusenda, and Kodrianka Valbusenda. **Bottom** row (left to right): Maturana Blanca IMIDRA 2020, Mollar Cano IMIDRA 2020, Muscat Hamburg IMIDRA 2020, Muscat Hamburg IMIDRA 2024, Prieto Picudo IMIDRA 2020, and Sabro IMIDRA 2020. Blue lines indicate the average outline of each population, whereas grey lines represent the individual seed outlines.

The 12 populations assigned to Group II are listed in Table 3. Among these, Beba, Mollar Cano, and Sabro IMIDRA 2020 had been assigned initially to the Hebén lineage group, whereas Kodrianka Valbusenda and Muscat Hamburg had originally been classified within the Muscat group. The remaining populations were initially assigned to the Savagnin Blanc group. Representative seed outlines of the Group II populations are shown in Figure 3.

**Figure 4:**
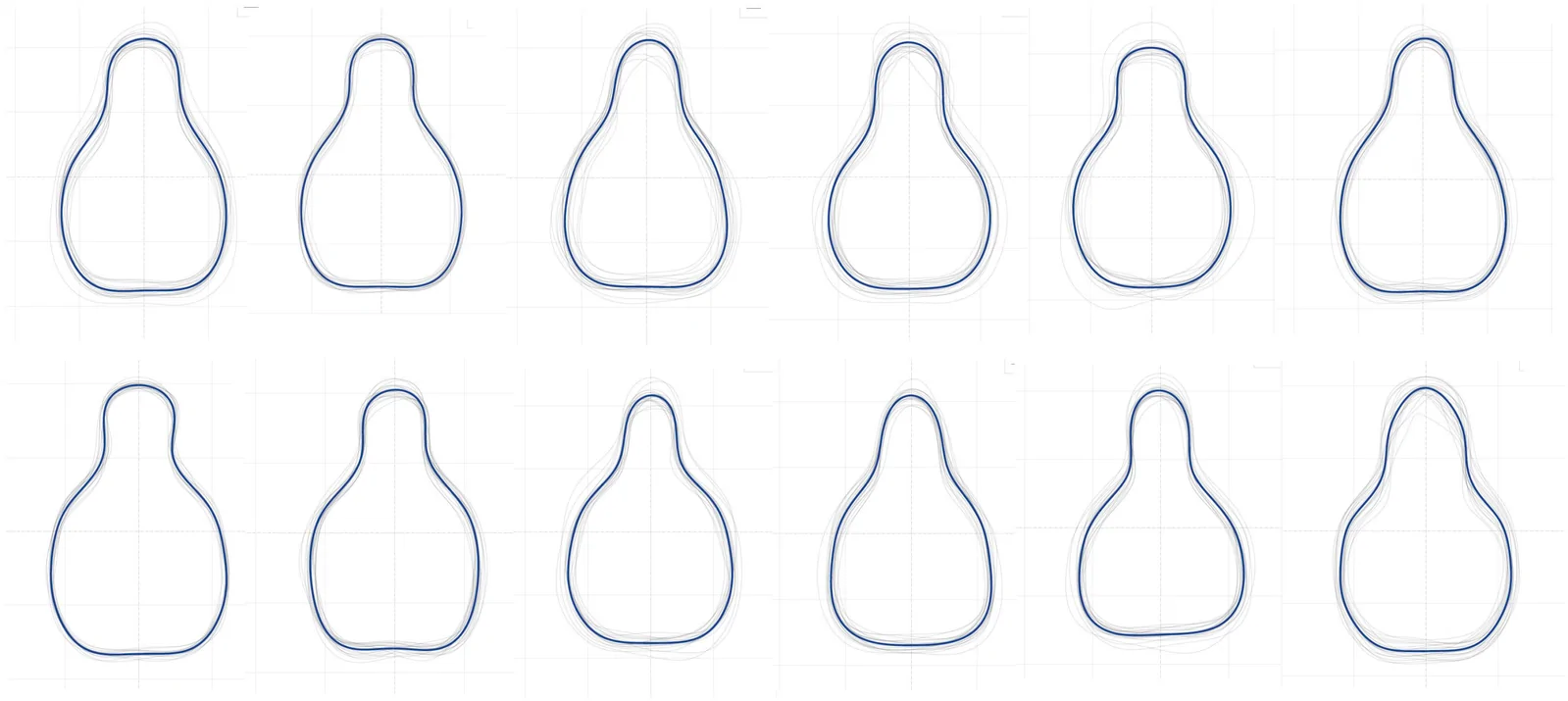
Average seed outlines of populations assigned to Group III (Muscat) following *J* index-based classification. **Top** row (left to right): Airén IMIDRA 2020, Arinto INIAV Pt, Bobal IMIDRA 2020, Borba IMIDRA 2020, Camarate Tinto INIAV Pt, and Cariñena Mazuela IMIDRA 2020. **Bottom** row (left to right): Chenin Blanc IMIDRA 2020, Gouveio INIAV Pt, Matilde Valbusenda, Mazuela Valbusenda, Muscat of Alexandria Valbusenda, and Parduca IMIDRA 2020. Blue lines indicate the average outline of each population, whereas grey lines represent the individual seed outlines.

The 21 populations assigned to Group III are listed in Table 4 and Supplementary Table S1 (Sheet 3). These populations originated from all three preliminary lineage groups: seven from the Muscat group (Borba, Cariñena Mazuela, Matilde, Mazuela, Muscat of Alexandria, and Red Globe IMIDRA and Valbusenda), five from the Hebén group (Airén, Bobal, Perrum, Trincadeira das Pratas, and Vijariego Blanco), and nine from the Savagnin Blanc group (Arinto, Camarate Tinto, Chenin Blanc, Gouveio, Parduca, Sauvignon Blanc, Verdejo, and Verdelho INIAV Pt 50317 and 51509). Representative average seed outlines of the Group III populations are shown in Figure 4.

**Figure 5:**
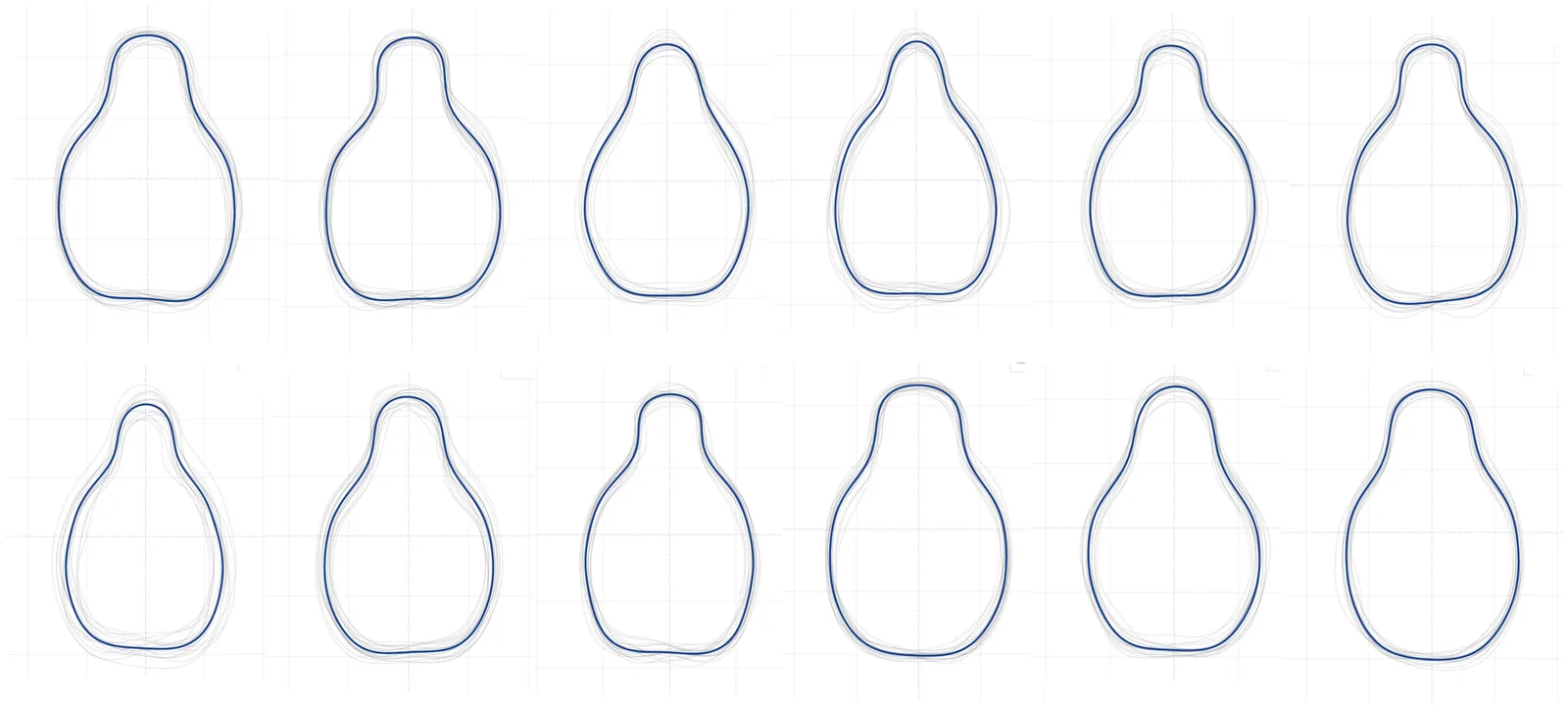
Average seed outlines of populations assigned to Group IVa (equally similar to the Hebén and Savagnin Blanc) following *J* index-based classification. **Top** row: Albillo Mayor Abadía Retuerta, Albillo Mayor IMIDRA, Alcañón IMIDRA, Aledo Valbusenda, Alfrocheiro Valbusenda, and Alphonse Lavallée IMIDRA. **Bottom** row: Alphonse Lavallée Valbusenda, Bastardo Negro IMIDRA, Budelho INIAV Pt, Cabernet Sauvignon Abadía Retuerta, and Ayuso IMIDRA. Blue lines indicate the average outline of each population, whereas grey lines represent the individual seed outlines.

**Figure 6:**
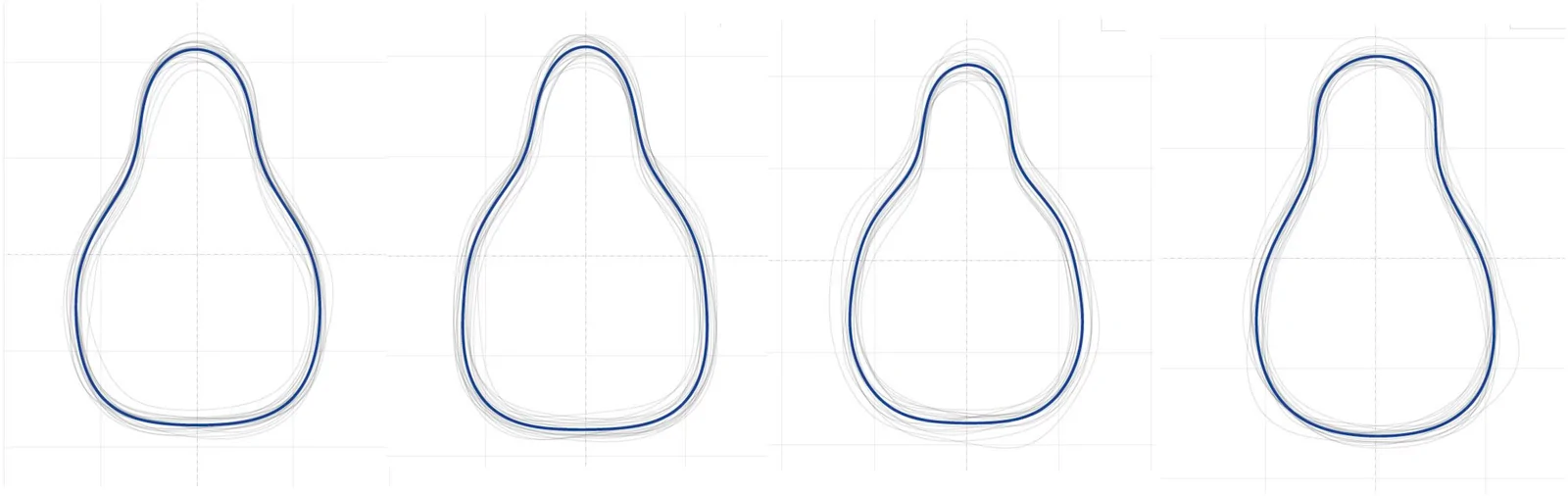
Average seed outlines of populations assigned to Group IVb (equally similar to the Hebén and Muscat reference models) following *J* index-based classification. From left to right: Airén Ayuso, Mazuela Abadía Retuerta, Muscat Hamburg Valbusenda, and Tarragoní IMIDRA 2024. Blue lines indicate the average outline of each population, whereas grey lines represent the individual seed outlines.

**Figure 7:**
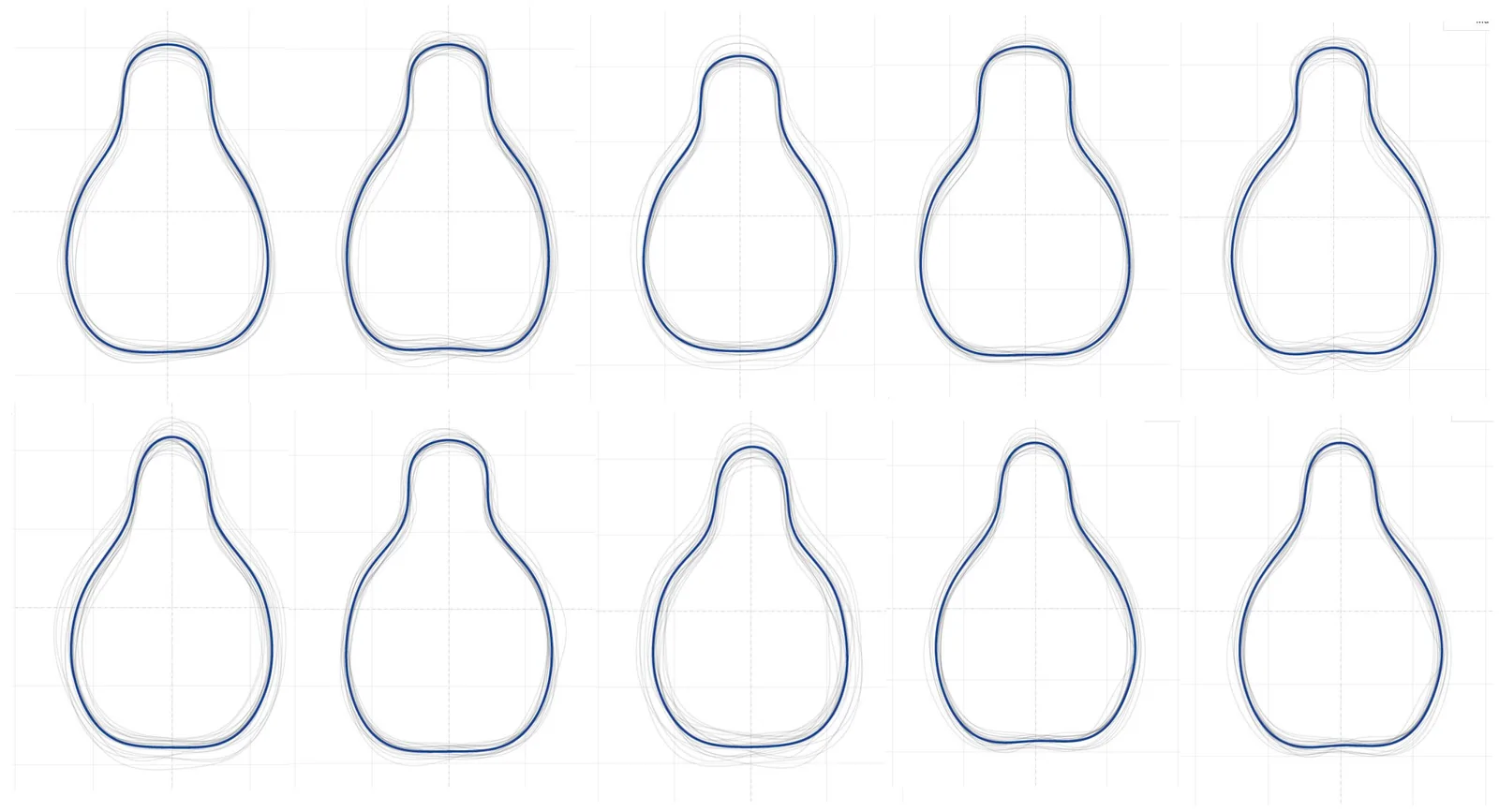
Average seed outlines of all populations assigned to Group V (equally similar to the three reference models) following *J* index-based classification. **Above** (left to right): Albillo de Granada IMIDRA 2020, Bastardo Espanhol INIAV Pt, Derechero IMIDRA 2020, Ferral IMIDRA 2020, and Folgasao Roxo INIAV Pt. **Below** (left to right): Koenigin der Weigarten IMIDRA 2020, Mollar Cano IMIDRA 2024, Moscato Ambrosio Valbusenda, Sauvignon Blanc IMIDRA 2020, and Sauvignon Blanc INIAV Pt. Blue lines indicate the average outline of each population, whereas grey lines represent the individual seed outlines.

A total of 59 populations exhibited comparable *J* index values for two reference morphotypes and therefore could not be assigned unambiguously to a single group. Of these, 55 showed similar affinity to the Hebén and Savagnin Blanc morphotypes and were assigned to Group IVa, whereas the remaining four exhibited comparable similarity to the Hebén and Muscat morphotypes and were assigned to Group IVb. An additional ten populations could not be distinguished from any of the three reference morphotypes or their combinations and were therefore assigned to Group V. Representative average seed outlines of Groups IVa, IVb, and V are shown in Figure 5, Figure 6 and Figure 7, respectively.

Figure 6 contains average outlines of populations of Group IVb.

The undifferentiated populations assigned to Group V were: Albillo de Granada IMIDRA 2020, Bastardo Espanhol INIAV Pt, Derechero IMIDRA 2020, Ferral IMIDRA 2020, Folgasao Roxo INIAV Pt, Koenigin der Weigarten IMIDRA 2020, Mollar Cano IMIDRA 2024, Moscato Ambrosio Valbusenda, Sauvignon Blanc IMIDRA 2020 and Sauvignon Blanc INIAV Pt (Figure 7).

### 2.3. Curvature Analysis in the Morphotypes

**Figure 8:**
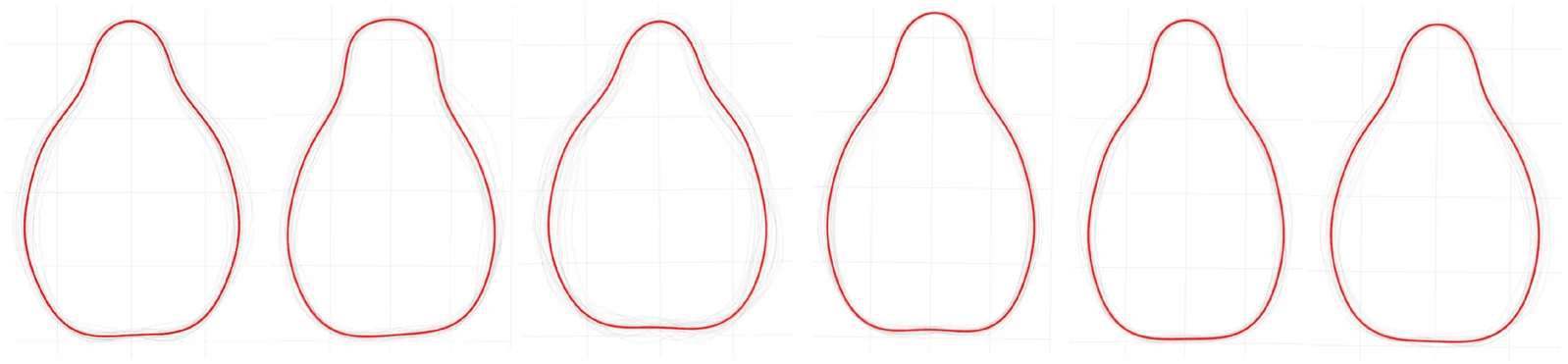
Average seed outlines of the six populations selected as representative of Group I (highest similarity to the Hebén reference morphotype). From left to right: Malvar IMIDRA 2020, Verdil IMIDRA 2020, Tinta de Toro Abadía Retuerta, Verdejo de Salamanca IMIDRA 2020, Xarello IMIDRA 2020, and Miguel de Arco IMIDRA 2024. Red lines indicate the average outline of each population, whereas grey lines represent the individual seed outlines.

**Figure 9:**
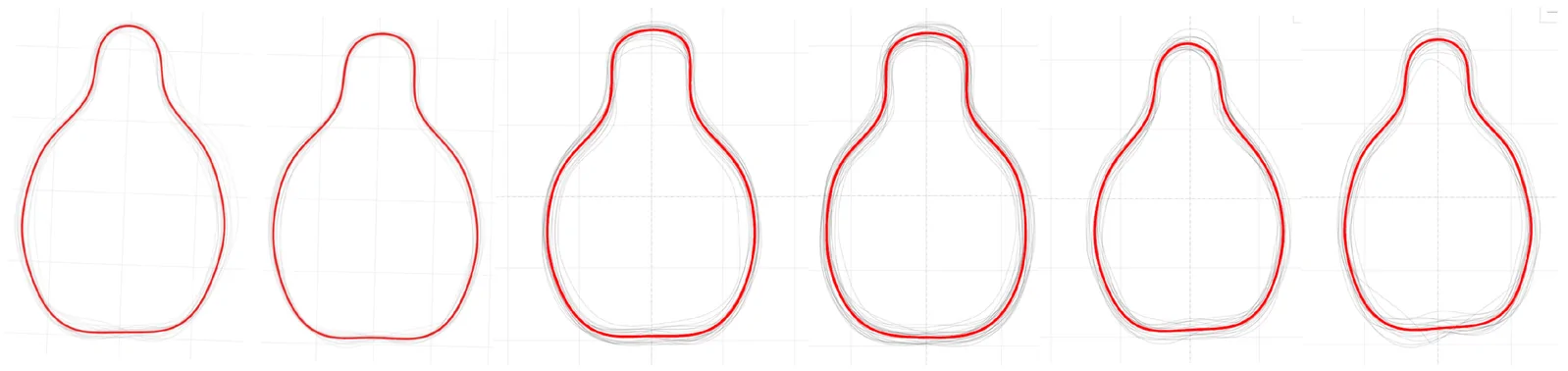
Average seed outlines of the six populations selected as representative of Group II (highest similarity to Model Savagnin Blanc). From left to right: Beba IMIDRA 2020, Alfrocheiro IMIDRA 2020, Maturana Blanca IMIDRA 2020, Prieto Picudo IMIDRA 2020, Kodrianka Valbusenda, and Albarín Blanco IMIDRA 2020. Red lines indicate the average outline of each population, whereas grey lines represent the individual seed outlines.

**Figure 10:**
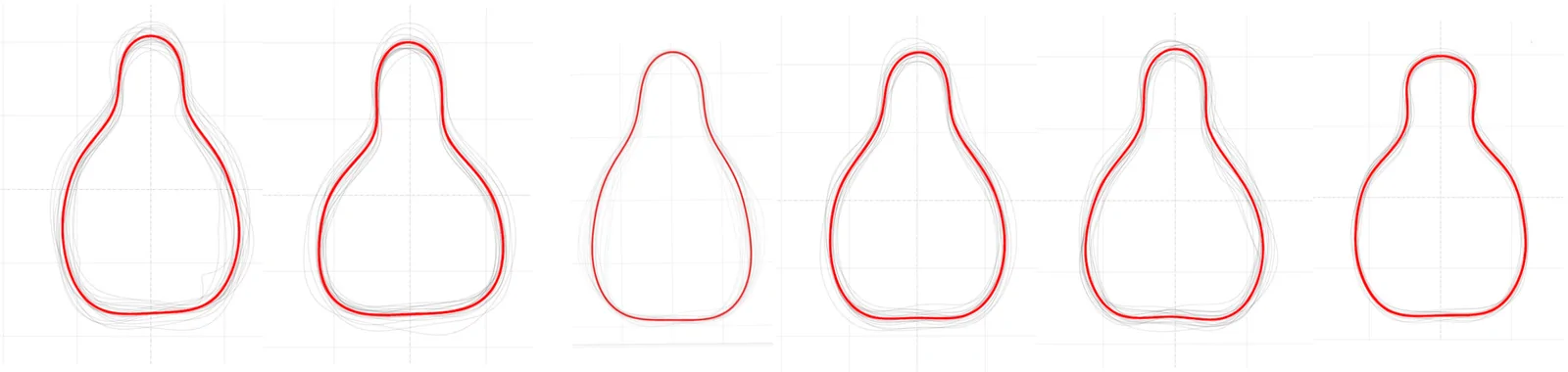
Average seed outlines of the six populations selected as representative of Group III (highest similarity to Model Muscat). From left to right: Sauvignon Blanc Abadía Retuerta, Muscat of Alexandría Valbusenda, Bobal IMIDRA 2020, Cariñena Mazuela IMIDRA 2020, Red Globe Valbusenda, and Chenin Blanc IMIDRA 2020. Red lines indicate the average outline of each population, whereas grey lines represent the individual seed outlines.

Curvature analysis was performed on the populations assigned to Groups I, II, and III following *J* index-based classification to quantitatively characterize the three principal seed morphotypes. After establishing Groups I, II, and III according to their similarity to the Hebén, Savagnin Blanc, and Muscat reference morphotypes, respectively, curvature values were calculated at the five characteristic landmark positions (P1–P5; Figure 1) for all seed outlines within each group. The six populations closest to the centroid of each morphometric group were subsequently selected using the centroid-based procedure described in the Materials and Methods. These populations were considered the most representative of their respective morphotypes and were used for the detailed curvature analyses presented below. Their average seed outlines are shown in Figure 8, Figure 9 and Figure 10 for Groups I, II, and III, respectively.

**Figure 11:**
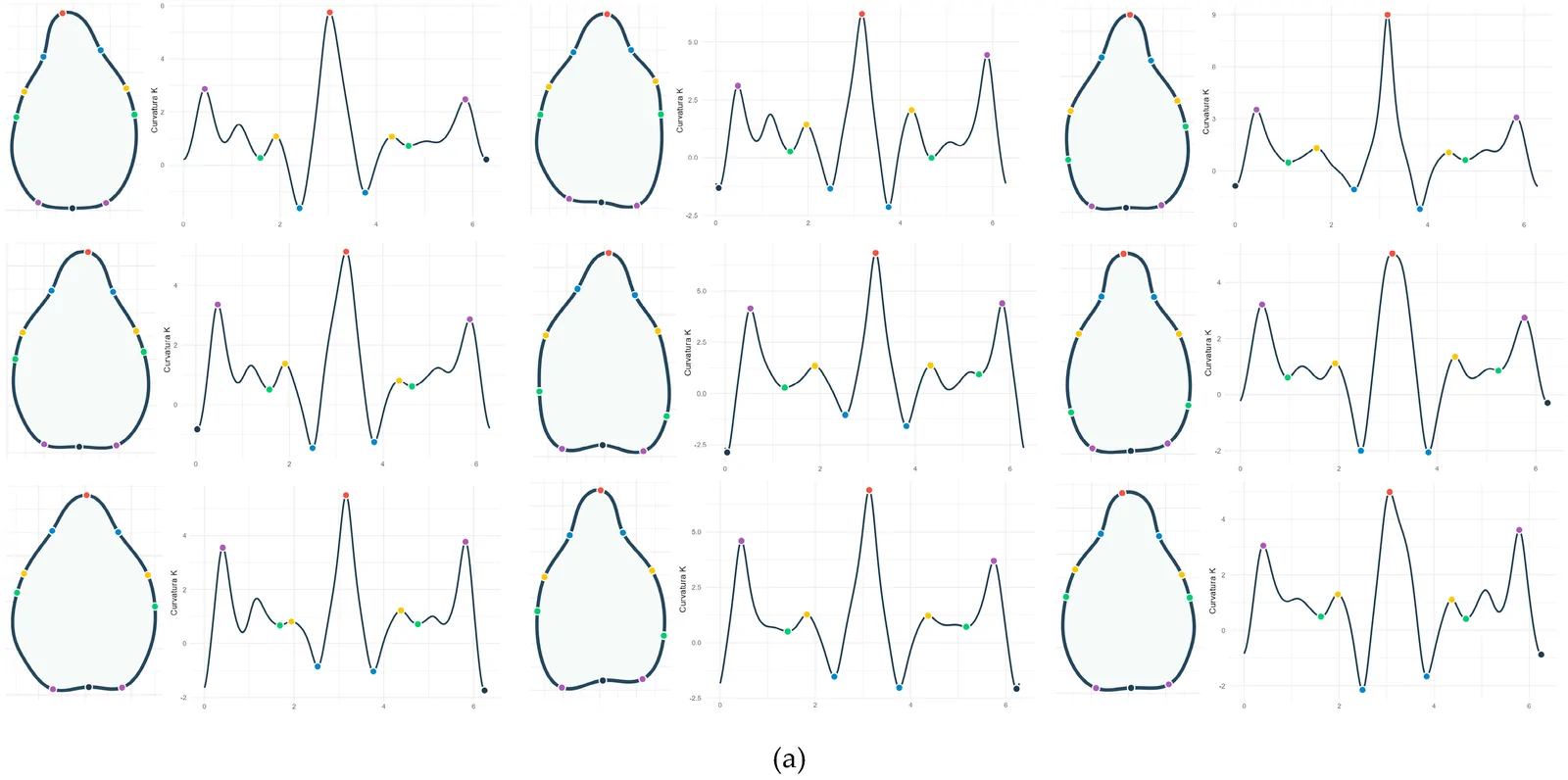

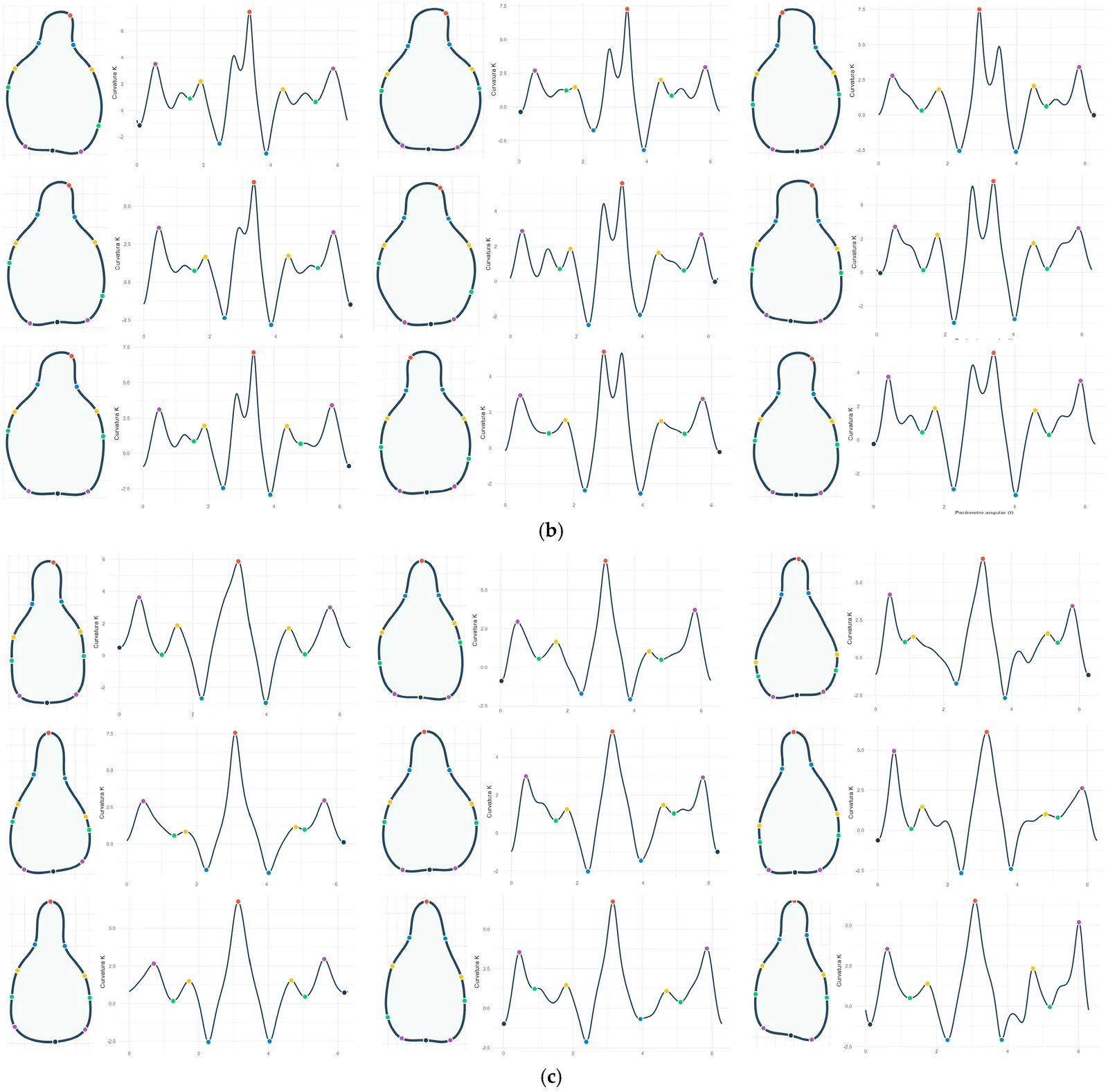
Curvature profiles of seed outlines representing the three principal grapevine morphotypes. (**a**) Group I (Hebén morphotype): **first column**, Malvar IMIDRA 2020; **second column**, Tinta de Toro Abadía Retuerta; **third column**, Verdejo de Salamanca IMIDRA 2020. (**b**) Group II (Savagnin Blanc morphotype): **first column**, Alfrocheiro IMIDRA 2020; **second column**, Maturana Blanca IMIDRA 2020; **third column**, Prieto Picudo IMIDRA 2020. (**c**) Group III (Muscat morphotype): **first column**, Muscat of Alexandria Valbusenda; **second column**, Cariñena Mazuela IMIDRA 2020; **third column**, Red Globe Valbusenda. For each sample, the seed outline and its corresponding curvature profile (curvature as a function of position along the seed outline) are shown. Characteristic curvature landmarks are indicated on each profile: red, P1; blue, P2 and P2′; yellow, P3 and P3′; green, P4 and P4′; and purple, P5 and P5′.

Figure 11 shows the curvature profiles of individual seeds corresponding to the outlines presented in Figure 8, Figure 9 and Figure 10.

Curvature values were calculated for all seed outlines assigned to Groups I, II, and III. Within each group, the six populations closest to the centroid of the corresponding morphometric cluster were selected as representative for comparative analysis. Their mean curvature values at the five characteristic landmarks are summarized in Table 5.

## 3. Discussion

### 3.1. Automated Identification of Curvature Landmarks

A major advantage of the automated workflow developed in this study is its ability to identify homologous curvature landmarks along grapevine seed outlines without manual intervention. The current implementation is based on five anatomically meaningful landmark positions (P1–P5), providing a reproducible and objective framework for describing seed geometry across cultivars.

This strategy proved robust across the large and morphologically diverse dataset analysed in the present study, enabling automated detection of the principal curvature landmarks required for quantitative comparisons among seed morphotypes. Nevertheless, the current implementation should be regarded as a flexible framework that can be refined as additional geometric criteria become available. For example, the apical region occasionally exhibited two closely spaced curvature maxima rather than a single dominant peak, a pattern observed in approximately 20% of the seeds and predominantly in seeds with rounded beaks. This observation suggests that supplementary decision rules or localized optimization procedures could further refine landmark identification in this region. Similar adjustments could be implemented for other portions of the seed outline where greater geometric precision is desirable. Owing to its modular design, the R workflow can readily accommodate such extensions while preserving the reproducibility, objectivity, and high-throughput capabilities of the method.

Because the procedure relies exclusively on local geometric properties of the reconstructed outline, homologous anatomical regions can be detected consistently across seeds with different shapes, allowing meaningful quantitative comparisons among populations and morphotypes. This level of automation minimizes observer bias, improves reproducibility, and makes the workflow suitable for high-throughput morphometric analyses of large germplasm collections.

### 3.2. Morphotypes and Haplotypes

Grapevine cultivars exhibit remarkable morphological diversity, reflecting thousands of years of domestication, repeated hybridization, vegetative propagation, and regional selection [37,38]. This diversity is evident not only in vegetative and reproductive organs but also in seed morphology, which has long been recognized as a valuable source of taxonomic and evolutionary information and has been widely applied in both ampelographic and archaeobotanical studies [23,24,25,26]. Quantitative morphometric analyses have further demonstrated that seed shape retains a substantial genetic component despite the considerable phenotypic variation observed among cultivars [28,29,30,31].

Despite this overall diversity, our results reveal a high degree of morphological consistency within the Hebén morphotype. Group I was the largest group identified in the present study and exhibited a characteristic seed architecture that was relatively conserved across populations. This observation agrees with previous studies identifying the Hebén morphotype as one of the principal seed morphotypes of cultivated *Vitis vinifera*, closely associated with Hebén and many of its documented descendants [10,23]. The pronounced morphological coherence observed within this group supports the interpretation that seed shape can preserve phenotypic signals associated with shared ancestry and can therefore provide information complementary to genomic data when investigating relationships among cultivars.

Several cultivars not currently recognized as descendants of Hebén nevertheless exhibited high *J* index values relative to the Hebén reference morphotype. At least two, not mutually exclusive, explanations may account for this observation. First, these cultivars may share previously unidentified ancestry with Hebén, reflecting the still incomplete reconstruction of historical grapevine pedigrees. Second, the Hebén morphotype may represent a recurrent developmental phenotype that has arisen independently in different genetic backgrounds, constituting a possible case of morphological convergence or homoplasy. Such convergence could result from similar developmental pathways or from repeated selection for particular seed architectures during grapevine domestication. Distinguishing between these alternatives will require integration of expanded pedigree information, haplotype-resolved genomic analyses, and quantitative morphometric data.

In contrast, the Savagnin Blanc morphotype exhibited lower morphological cohesion than the Hebén group. Several cultivars assigned to this morphotype are genetically related to Hebén, suggesting that this seed architecture may represent an intermediate phenotype rather than a strictly lineage-specific morphological signature. This interpretation is consistent with the extensive recombination that characterizes cultivated grapevine and indicates that not all seed morphotypes retain the same degree of correspondence with genetic lineages. Consequently, whereas the Hebén morphotype appears to provide a relatively consistent phenotypic indicator of shared ancestry, the Savagnin Blanc morphotype may encompass a broader range of developmental and evolutionary trajectories. These observations further illustrate the complementary nature of morphometric and genomic approaches for investigating grapevine diversification.

A contrasting pattern emerged for the Muscat morphotype. Whereas the Hebén lineage, and to a lesser extent the Savagnin Blanc lineage, retained recognizable seed architectures associated with founder lineages, the Muscat morphotype appeared to represent a more heterogeneous phenotypic group rather than the signature of a single ancestral cultivar. This morphotype occurred across a broad range of genetically diverse cultivars, many of which produce large berries and are widely cultivated as table grapes. Compared with the relatively conserved seed architecture of the Hebén group, the Muscat group displayed greater morphological variation and generally lower bilateral symmetry [39]. In *Medicago sativa* L., domesticated plants differ from their wild relatives, and deviations from perfect symmetry in leaves have been associated with hybridization and introgression [40]. Together, these observations suggest that the Muscat morphotype may reflect multiple developmental trajectories associated with domestication and selection rather than the persistence of a single founder-derived seed architecture. From a developmental perspective, strong human selection for increased flesh-to-berry ratio and larger fruit volume in table grapes may impose spatial constraints on developing seeds within the locular cavity, altering the physical pressure exerted by the surrounding pericarp. Furthermore, this physical co-evolution between seed size and pulp volume is tightly regulated by plant growth regulators [41,42]. Auxins and gibberellins synthesized by developing seeds drive pericarp cell division and expansion, while reciprocal maternal signalling modulates seed coat growth and sclerification [43,44]. Selection for larger berry size could thus perturb endogenous hormonal dynamics, leading to altered endocarp patterning, reduced bilateral symmetry, and the convergence toward a more rounded, compact seed architecture across phylogenetically distant lineages. Future studies integrating morphometric, developmental, and haplotype-resolved genomic data will be essential to test this hypothesis and to clarify the evolutionary processes underlying the emergence of the principal grapevine seed morphotypes.

The three primary reference morphotypes examined here—Hebén, Savagnin Blanc, and Muscat—were selected because they are associated with major founder lineages that have contributed substantially to European grapevine germplasm. Rather than defining a closed or exhaustive typology, these three morphotypes function as geometric anchors within a continuous phenotypic morphospace. The structure of this morphospace is particularly evident in the Muscat group, which exhibited lower internal morphological cohesion than the Hebén and Savagnin Blanc groups. The broad genetic and historical diversity encompassed by the Muscat group may contribute to this elevated variation. Consequently, the correspondence observed between seed morphotypes and genomic lineages should be interpreted as phenotypic associations with documented haplotypic lineages rather than as direct genetic validation. The ability of curvature analysis to capture this internal heterogeneity further indicates that the framework can represent biological variation rather than imposing artificial uniformity on the dataset.

### 3.3. Defining Archetypal Morphotypes by Centroid-Based Multivariate Analysis

Morphometric studies frequently involve the analysis of large biological collections in which substantial variation exists among specimens within the same group. Consequently, identifying a limited number of representative specimens or populations that accurately summarize the morphology of an entire group has become an important objective in geometric morphometrics. The representation of biological variation within a multivariate morphospace is a central concept of geometric morphometrics, in which distances between specimens and group centroids provide objective measures of morphological similarity [45,46]. In the present study, the morphospace was defined by curvature measurements at five homologous landmarks, allowing representative populations to be identified objectively according to their Euclidean distance from the centroid of each morphotype.

Centroid-based selection has been applied across a wide range of biological disciplines, including archaeobotany, plant systematics, medical imaging, and evolutionary morphology. For example, geometric morphometric analyses of olive endocarps have used multivariate shape descriptors to reconstruct patterns of domestication and historical biogeography [47]. Comparable distance-based approaches have also been employed to define representative anatomical configurations in medical image analysis [48] and to characterize morphological variation during vertebrate evolution [49]. In plant systematics, quantitative morphometrics has long provided an effective framework for describing morphological variation within clonally propagated crops while minimizing observer bias [50]. Extending these approaches, the present study demonstrates that curvature-derived morphospaces can provide an objective basis for defining representative grapevine seed morphotypes.

Representative populations were selected according to their Euclidean distance from the centroid of each morphometric group, as defined by curvature values at the five homologous landmarks. This procedure identified the six populations located closest to the multivariate centroid of each morphotype and therefore best representing its central morphological tendency while minimizing the influence of atypical or extreme phenotypes. Rather than relying on subjective visual selection, this approach provides a reproducible and quantitatively defined criterion for identifying archetypal morphotypes suitable for subsequent comparative analyses. In this context, an archetypal morphotype is defined here as the observed phenotype located closest to the centroid of a multivariate morphospace, representing the central tendency of a biological group rather than an extreme or idealized form. Thus, centroid-based selection provides a principled means of reducing complex within-group variation to a limited set of representative populations while retaining the principal morphological characteristics of each group. Automated morphometric classification can therefore serve as a valuable complement to genomic characterization in grapevine germplasm collections.

Although the workflow presented here was developed for grapevine seeds, the underlying strategy is broadly applicable to other biological structures that can be characterized using homologous morphometric variables. The centroid-based framework consequently provides a general methodology for identifying representative morphotypes within large biological datasets, facilitating standardized comparisons among studies, laboratories, and species. More broadly, combining automated morphometric analyses with genomic information offers a promising avenue for integrating phenotypic and genetic diversity, thereby helping to bridge the persistent gap between high-throughput genomics and quantitative phenotyping in plant biology.

### 3.4. Unassigned Morphotypes and Future Directions

The emergence of distinct unassigned morphometric groups (IVa, IVb, and V) does not necessarily imply a lack of affinity to the reference models. Rather, these groups comprise seeds exhibiting similar *J*-index values for two or three reference morphotypes, indicating overlapping morphometric affinities that cannot be resolved into a single category. The fact that 55 cultivars were equally similar to Hebén and Savagnin Blanc indicates a particularly close morphometric relationship between these two reference morphotypes. Nevertheless, their seed geometries remain distinguishable in specific features: lower seed solidity is associated with higher absolute curvature values at landmark $P_{2}$, a characteristic feature of the Savagnin Blanc morphotype. These results illustrate the complexity of grapevine seed diversity beyond the primary reference morphotypes and highlight the value of combining global contour similarity with local geometric descriptors.

This complexity also illustrates both the strengths and limitations of the computational framework. The *J* index provides an automated and reproducible measure of overall contour similarity, enabling objective macro-classification of large germplasm collections. However, global contour similarity alone may be insufficient to resolve morphotypes with overlapping geometries or complex combinations of parental characteristics. Integrating local boundary curvature provides additional discriminatory information by capturing fine-scale geometric features that may be masked by overall shape similarity. The complementary use of global and local descriptors therefore reduces the risk of interpreting a single similarity metric as a definitive morphometric classification.

To contextualize these findings within established ampelographic practice, it is important to clarify how automated seed morphometry relates to conventional phenotypic characterization systems such as the descriptor list of the International Organisation of Vine and Wine (OIV). Traditional cultivar identification relies on a broad suite of macroscopic descriptors covering leaves, shoots, inflorescences, bunches, and berries, whereas seed descriptors constitute only a small component of the overall characterization framework. Whole-plant ampelography therefore remains indispensable for comprehensive cultivar identification. However, many vegetative and fruit traits are phenotypically plastic and may vary according to climate, soil conditions, developmental stage, and agronomic management. In contrast, the mature grapevine endocarp provides a comparatively stable morphological structure and can retain diagnostic information in historical and archaeobotanical material in which soft tissues are no longer available. Standard OIV seed descriptors (OIV 241–244) [20] provide useful basic measurements and qualitative assessments, but their relatively low dimensionality may limit their ability to resolve subtle differences among closely related morphotypes. The automated EFA and curvature workflow presented here does not seek to replace multi-organ ampelography; rather, it extends conventional seed characterization by providing continuous, high-dimensional geometric descriptors. In this sense, the methodology complements the OIV framework and provides a scalable phenotypic approach connecting traditional ampelography with archaeobotany and genomic characterization.

A deliberate feature of the present framework was the focus on external two-dimensional outline geometry, using EFA and boundary curvature, rather than internal seed-surface features such as the chalaza pattern. Although the chalaza constitutes an established descriptive character in classical ampelography, its automated extraction presents additional computer-vision challenges. Internal surface features can be strongly affected by illumination, pigmentation, surface texture, and local shading, potentially reducing segmentation reproducibility in high-throughput applications. They may also be partially obscured or degraded in weathered or archaeological specimens, whereas the external dorsal and ventral contours generally provide a more robust geometric boundary. Focusing on continuous boundary descriptors therefore maximizes automation, reproducibility, and applicability across heterogeneous germplasm collections. Nevertheless, internal seed characters should not be regarded as biologically uninformative. Future developments incorporating three-dimensional surface scanning, multispectral imaging, or convolutional neural network (CNN)-based image segmentation could potentially integrate chalaza position, depth, and other internal anatomical features as complementary descriptors.

The practical scope and resolution limits of automated seed morphometry should therefore be clearly defined. Rather than functioning as a standalone method for definitive individual cultivar identification, the present framework is best considered a high-throughput complementary descriptor that extends conventional seed ampelography. When applied to a newly characterized landrace or breeding accession, the workflow can provide a rapid and objective phenotypic baseline by quantifying both global contour similarity through *J*-index fitting and local geometric variation through boundary curvature. This may facilitate preliminary assessment of morphometric affinity to major reference groups without requiring prolonged multi-season evaluation of vegetative and reproductive characters. Such an approach could be particularly useful for germplasm repositories and breeding programmes as a first-pass screening tool for large collections.

However, the biological resolution of seed morphometry has clear limits. Although the method can distinguish major morphotypes and groups of seeds with contrasting geometric profiles, should not be expected to reliably discriminate among closely related cultivars that share similar seed-parent architecture. Seed endocarp shape is influenced by polygenic developmental processes and by the mechanical constraints imposed during seed formation within the berry; consequently, substantial phenotypic overlap among genetically related cultivars is biologically plausible. The unassigned groups identified in the present study should therefore be regarded as morphometrically informative categories rather than as evidence of discrete genetic lineages. Determining whether these groups correspond to particular ancestral backgrounds, recurrent hybrid combinations, or developmental variants will require their integration with independent genetic evidence.

Future research should consequently expand the morphometric reference space by incorporating additional *V. vinifera* landraces, historical cultivars, wild *V. vinifera* subsp. *sylvestris*, and genetically characterized breeding material. Combining the present automated workflow with high-density SNP genotyping and haplotype-resolved genomic data could then allow specific seed-shape features to be associated with genetic ancestry and, ultimately, with quantitative trait loci (QTLs) underlying seed morphogenesis. Such integration would provide a direct framework for testing whether the morphometric groups identified here correspond to haplotypic backgrounds and for determining the extent to which seed morphology reflects ancestry, developmental constraints, and environmental variation. In this context, automated seed morphometry should be viewed not as a replacement for genomic or classical ampelographic approaches, but as a complementary quantitative phenotype that can help connect genotype, morphology, and grapevine diversity across modern, historical, and archaeological collections.

## 4. Materials and Methods

### 4.1. Plant Material and Sampling Strategy

A total of 2895 seeds from 143 populations representing 85 *Vitis vinifera* cultivars were analysed. The complete germplasm collection, including cultivar names, geographical provenance, accession codes, pedigree information, and initial genetic classification, is provided in Supplementary Table S1 (Sheet 1) (see Supplementary Materials).

Seeds were collected from berries located at three distinct positions within the cluster: the region near the peduncle, the central region, and the region opposite the peduncle. Prior to analysis, seeds were visually inspected to ensure structural integrity and to exclude physically deformed or damaged specimens. This sampling strategy provided sufficient material to select 20 intact, well-developed seeds per population. A sample size of 20 seeds per population was selected based on established sampling practices in *Vitis* geometric morphometrics [22]. This sample size was considered adequate for characterizing within-population variation using Elliptic Fourier Analysis (EFA) and boundary-curvature profiling.

To evaluate both within-cultivar morphological stability and environmental variation, seed samples were collected from multiple populations representing different cultivation sites and sampling years. This sampling strategy enabled the assessment of morphological consistency among populations of the same cultivar while accounting for variation associated with growing conditions and sampling year.

The germplasm originated from two main sources:

Institutional germplasm repositories: Instituto Madrileño de Investigación y Desarrollo Rural, Agrario y Alimentario (IMIDRA, Madrid Spain), sampled in 2020, 2024, and 2025, and Instituto Nacional de Investigação Agrária e Veterinária (INIAV, Oeiras, Portugal), sampled in 2006.

Commercial vineyards and wine estates: Abadía Retuerta, Valbusenda, Bodegas Ayuso, Divina Proporción, and Liberalia, sampled in 2025.

### 4.2. Assignment of Structural Morphotypes

To provide an evolutionary framework for the morphometric analyses, cultivars were initially assigned to three major structural morphotype groups based on published genomic and pedigree information. The primary classification followed the SNP haplotype groups identified by [27] and subsequently incorporated into geometric morphometric analyses of grapevine seed shape [23,28,29,30,31]. Pedigree relationships were compiled from established reconstructions of grapevine ancestry [5,6,8,9,10,36]. This preliminary classification was used exclusively as an initial biological hypothesis to define the reference groups. Final morphometric assignments were subsequently determined independently using the automated *J* index procedure described below.

The preliminary classification divided the germplasm into three principal lineage groups (Supplementary Table S1, Sheets 1 and 2):

Hebén group (Haplotype 5): Traditional Iberian cultivars centred on Hebén and its documented descendants. This group comprised 48 cultivars, represented by 80 populations and 1636 seeds.

Savagnin Blanc group (Haplotype 6): Central European cultivars represented by Savagnin Blanc and related lineages. This group comprised 22 cultivars, represented by 35 populations and 715 seeds.

Muscat group (Haplotype 3): Cultivars related to Koenigin der Weingarten and the Muscat lineage. This group comprised 15 cultivars, represented by 28 populations and 544 seeds.

### 4.3. Seed Images

The seed images analysed in this study were obtained from previously published datasets generated in our laboratory [23,28,29,30,31,51]. The original images were acquired under standardized imaging conditions and are publicly available, together with their associated metadata, through the repositories listed in the Supplementary Materials.

### 4.4. Elliptic Fourier Descriptors (EFDs)

Seed outlines were extracted from binary images and mathematically described using Elliptic Fourier Analysis (EFA) implemented in the R package Momocs [52]. Each closed outline was represented by a series of Elliptic Fourier Descriptors (EFDs), consisting of numerical Fourier coefficients that provide a normalized mathematical representation of seed shape. The normalization procedure removes the effects of translation, rotation, scaling, and, where applicable, the position of the starting point along the contour, thereby allowing seed outlines to be compared independently of their size, orientation, and position. The mathematical formulation of EFA and the normalization procedure have been described in detail elsewhere [29,31].

Each harmonic (*n*) is defined by four Fourier coefficients (*A*_n_, *B*_n_, *C*_n_, and *D*_n_), which collectively describe the contribution of that harmonic to the geometry of the outline. Lower-order harmonics capture broad aspects of seed shape, including overall elongation, width, and general contour geometry, whereas higher-order harmonics progressively capture finer-scale features, such as apex sharpness, notch depth, and localized basal or shoulder curvature. The EFDs were extracted automatically using the R package Momocs [52].

As the number of harmonics increases, the reconstructed parametric curve progressively approximates the original biological outline. Following normalization, the resulting descriptors provide standardized representations of shape that are directly comparable among samples. The reconstructed outlines were subsequently used as the basis for additional geometric analyses. Among these, the *J* index quantified the similarity between a reconstructed seed outline and a predefined geometric reference model, providing an objective measure of morphotype affinity. Complementary to this global shape descriptor, boundary-curvature analysis quantified local geometric variation along the seed outline and enabled the identification of diagnostically informative features. Together, these approaches combined global contour similarity with localized geometric information, providing a comprehensive framework for quantitative seed-shape characterization. In the present study, both descriptors were incorporated into a fully automated computational workflow for the high-throughput analysis of *Vitis vinifera* germplasm.

### 4.5. Computational Reconstruction of Seed Outlines from Elliptic Fourier Descriptors

Normalized EFDs were used to reconstruct the two-dimensional outlines of grapevine seeds. The number of Elliptic Fourier harmonics was set to *n* = 8, corresponding to 32 normalized Fourier coefficients per outline. This parameter choice was supported by previous morphometric analyses of *Vitis* seed outlines [34], which showed that seven harmonics captured more than 95% of the total outline shape variation. The use of eight harmonics therefore provided an additional level of contour resolution while retaining the major geometric characteristics of the seed outline and avoiding the excessive inclusion of higher-order harmonics that may be more susceptible to image discretization and boundary noise.

Seed outlines were reconstructed using the inverse parametric equations of Elliptic Fourier Analysis. The x- and y-coordinates of the contour were calculated as functions of the cumulative curvilinear parameter t according to

$$x(t)=\sum_{n=1}^{N}\left(A_{n}cos\;nt+B_{n}sin\;nt\right)$$

$$y(t)=\sum_{n=1}^{N}\left(C_{n}cos\;nt+D_{n}sin\;nt\right),$$

where N denotes the number of harmonics included in the reconstruction. In the present study, eight harmonics (N = 8) were used for all analyses, providing an accurate representation of grapevine seed outlines while maintaining computational efficiency.

The contour was reconstructed by the evaluation of the Fourier series at 500 equally spaced values of t over the interval [0, 2π], obtaining a closed polygon suitable for subsequent geometric analyses. The reconstructed outlines preserved the normalized shape information encoded in the EFDs and served as the basis for all subsequent *J* index calculations and curvature analyses.

The reconstruction algorithm was implemented in R to enable the rapid and reproducible processing of large numbers of seed outlines while minimizing computational time. The resulting high-resolution parametric outlines provided a common geometric framework for all subsequent morphometric analyses.

Representative reconstructed outlines are shown in Figure 3, Figure 4, Figure 5, Figure 6, Figure 7, Figure 8, Figure 9 and Figure 10, in which the average outline of each population (blue or red) is superimposed on the outlines of the individual seeds (thin gray lines) used to calculate the population mean.

### 4.6. Computational Implementation

#### 4.6.1. Computational Workflow

**Figure 12:**
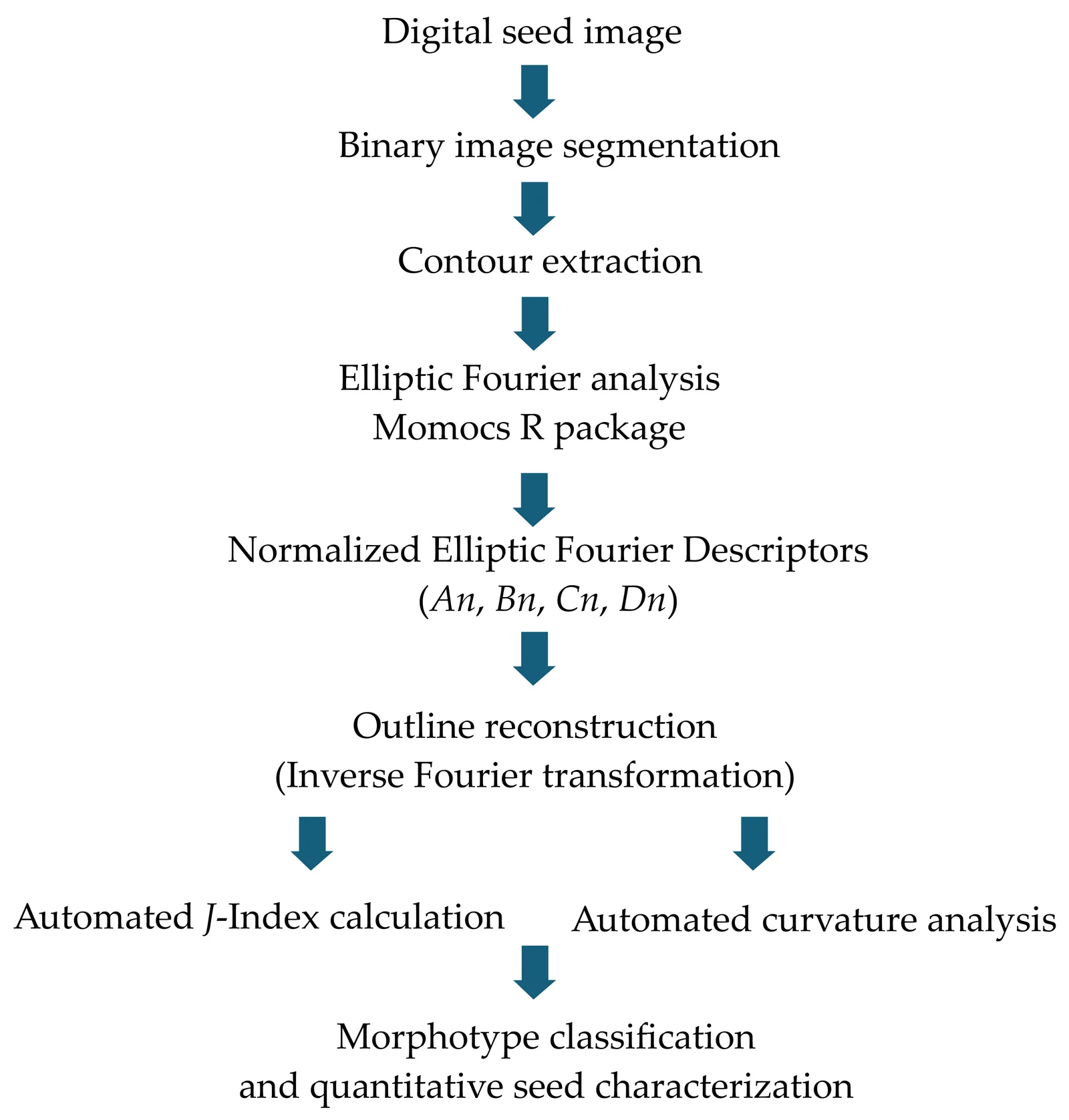
Schematic representation of the automated computational workflow developed for the quantitative analysis of grapevine seed morphology. Digital seed images were first converted into binary images by segmentation, after which the seed contours were extracted. The extracted contours were mathematically described using Elliptic Fourier Analysis (EFA), yielding a set of normalized Elliptic Fourier Descriptors (EFDs; coefficients A_n_, B_n_, C_n_, and D_n_). These descriptors were subsequently used to reconstruct complete seed outlines by inverse Fourier transformation. The reconstructed outlines provided the common geometric framework for two complementary analyses: (i) automated calculation of the *J* index, which quantifies the percentage similarity between each seed and predefined reference morphotypes, and (ii) automated curvature analysis, which identifies homologous curvature landmarks and quantifies local geometric variation along the seed boundary.

The complete computational workflow is summarized in Figure 12. Digital seed images were first converted into binary silhouettes, from which closed outlines were extracted. These outlines were mathematically described using Elliptic Fourier Analysis (EFA), yielding normalized Elliptic Fourier Descriptors (EFDs) that provided a compact representation of seed geometry. Inverse Fourier transformation was subsequently used to reconstruct each outline from its Fourier coefficients, generating a high-resolution parametric representation of the seed contour.

The reconstructed outlines then provided the common geometric framework for two complementary analyses. First, they enabled automated calculation of the *J* index, which quantified the percentage similarity between each seed outline and the predefined reference morphotypes. Second, they provided the continuous coordinate sequences required for automated curvature analysis, allowing the identification of homologous curvature landmarks and the quantitative characterization of local geometric variation along the seed boundary. Together, these procedures constituted a fully automated and reproducible pipeline for the high-throughput morphometric characterization of grapevine seeds.

#### 4.6.2. Computational Performance and Multi-Stage Pipeline Execution

To evaluate the scalability of the automated framework, computational benchmarks were recorded on a standard desktop workstation equipped with an 8-core processor (2.90 GHz base clock) and 16 GB of RAM and running R version 4.3.0. The analytical workflow comprised four sequential execution modules applied to all samples (Figure 12):

1. EFD Extraction: image binarization, contour tracing, and extraction of normalized Elliptic Fourier Descriptors.
2. *J* Index calculation: automated geometric comparison and alignment of reconstructed seed outlines with the predefined reference model shapes.
3. Morphotype classification: assignment of populations to reference or intermediate morphotype groups according to their morphometric similarity profiles.
4. Curvature analysis: localized evaluation of boundary curvature and automated identification of apical and other homologous curvature landmarks.

Processing speed. The *J* index calculation represented the principal computationally intensive stage of the workflow because each reconstructed seed outline had to be compared and aligned with the reference models.

Execution failures. Failures occurred predominantly during Module 1 when dust particles or other image artifacts altered the detected seed boundary and consequently affected contour tracing.

Exclusion criteria. Seeds identified as damaged, structurally incomplete, or affected by segmentation artifacts were systematically excluded from downstream analyses before model fitting. No manual editing of individual outline coordinates was performed. This procedure minimized operator intervention and maintained the objectivity and reproducibility of the computational workflow.

### 4.7. J Index Calculations

The *J* index represents the percentage similarity between a biological seed outline and a predefined reference morphotype. In the present study, an automated workflow implemented in R was developed to reconstruct seed outlines directly from their normalized Elliptic Fourier Descriptors (EFDs) and calculate *J* index values relative to each reference model.

The computational workflow was developed by the authors with programming assistance from Gemini v. 5.0 (Google) and is provided in full in the Supplementary Materials to facilitate reproducibility and reuse. The scripts were manually reviewed, tested, and validated by the authors before their application to the complete dataset.

Briefly, the algorithm reconstructs the outline of each seed from its normalized Fourier coefficients using the inverse Elliptic Fourier transformation. Each reconstructed outline is then geometrically aligned with a reference morphotype, and the proportion of overlapping area between both outlines is calculated. The resulting overlap, expressed as a percentage, constitutes the *J* index, with higher values indicating greater morphological similarity to the reference model.

$$J=100\times \frac{{a(A}_{R}\cap A_{M})}{{a(A}_{R}\cup A_{M})}$$

where *A_R_* is the reconstructed seed outline and $A_{M}$ is the reference model and *a*(*S*) denotes the area of a region *S*. This fully automated procedure enables the rapid, objective, and reproducible comparison of thousands of seed outlines without manual intervention. By replacing subjective visual comparisons with a standardized geometric metric, the method provides a framework for morphometric classification of grapevine cultivars and quantitative evaluation of seed morphotypes.

### 4.8. Curvature Analysis

**Figure 13:**
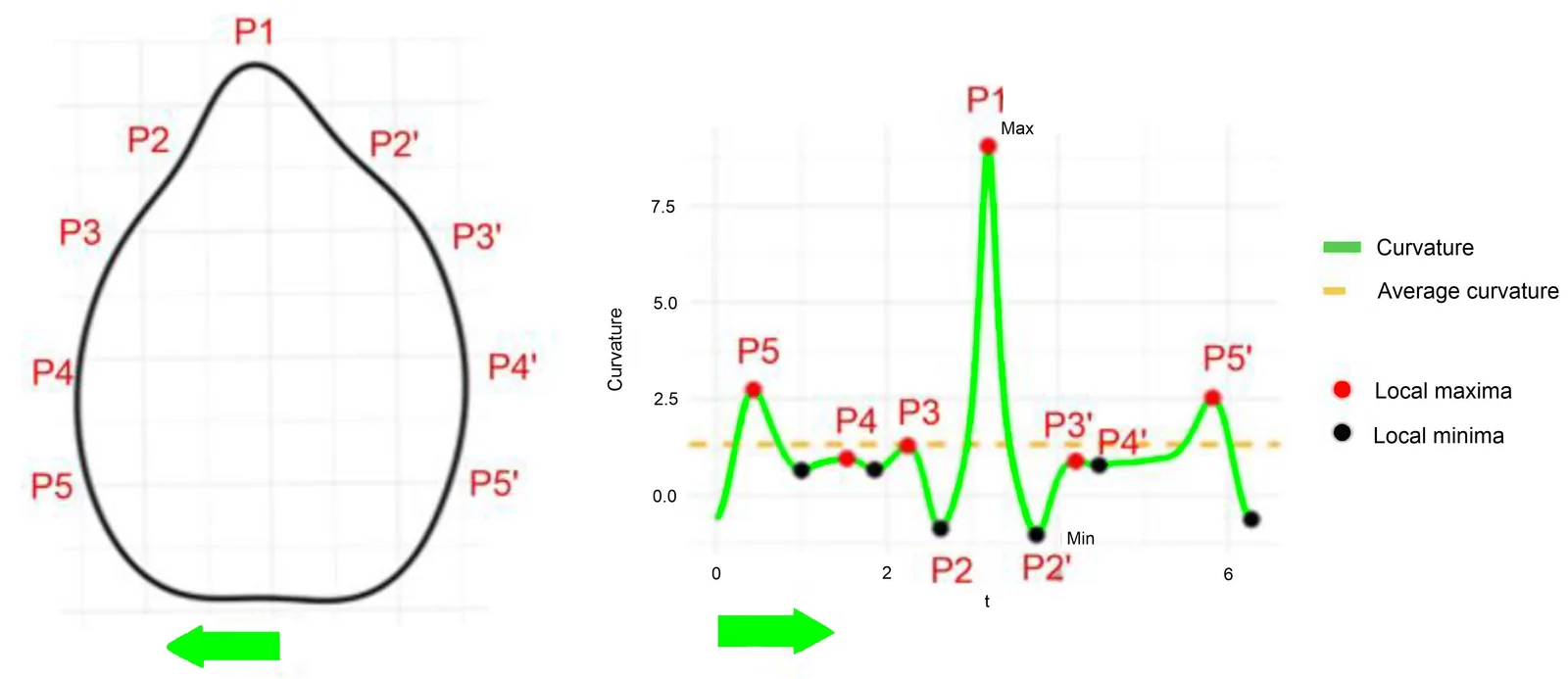
Automated identification of landmarks and quantification of curvature on reconstructed grapevine seed outlines. **Left**: Reconstructed outline of a *Vitis mustangensis* seed obtained from an Elliptic Fourier representation using eight harmonics. The numbered landmarks (P1–P5 and their corresponding points on the opposite side of the seed) indicate the characteristic curvature positions identified automatically by the algorithm. **Right**: Curvature profile calculated along the seed outline. Positive peaks (red) correspond to local curvature maxima, whereas black points indicate local minima. Landmark P1 represents the maximum curvature at the seed apex, while P2 and P2′ correspond to the negative curvature minima located in the chalaza–beak transition notch. The dashed orange line indicates the average curvature calculated along the entire contour. In this example, Max = 9.06, Min = −1.03, and average curvature = 1.31. Green arrows indicate the origin of the contour and the direction in which the boundary is traversed during curvature calculation. *Vitis mustangensis* was selected for this Figure to demonstrate the algorithmic robustness of our automated landmarking procedure on challenging outline variations.

A total of 1441 seeds belonging to 74 populations were assigned by the *J* index procedure to Groups I (41 populations), II (12 populations), and III (21 populations). Curvature analysis was subsequently performed on the reconstructed seed outlines of these 74 populations by the method represented in Figure 13.

Curvature was calculated from the continuous parametric representation of each reconstructed outline using an automated workflow implemented in R [53], adapted from the algorithm originally developed by Tocino [35]. Coordinates along each boundary contour were sampled to construct parametric Bézier curves representing the seed outline. Local curvature was then calculated continuously along the fitted Bézier curve as the rate of change in the tangent direction with respect to arc length. This procedure generated a continuous curvature function along the seed boundary, from which the mean curvature values and local curvature maxima and minima could be identified. These features provided the geometric basis for the objective identification of homologous landmarks and the quantitative comparison of seed morphotypes.

The algorithm first identified the pair of curvature minima (P2 and P2′) located within the chalaza–beak transition region. Because these minima correspond to a distinctive feature of the grapevine seed outline, they were used as reference points for the subsequent identification of the remaining landmarks. Following identification of the P2/P2′ transition region, the algorithm identified the point of maximum positive curvature at the apex of the seed beak (P1), followed by three successive pairs of landmarks in the chalaza region (P3/P3′, P4/P4′, and P5/P5′). These landmark pairs correspond, respectively, to the transition between the minimum-curvature region and the region of maximum chalaza breadth, the points of maximum seed width, and the limits of the basal region of the seed.

The complete procedure was fully automated, ensuring consistent and reproducible identification of homologous curvature landmarks across the analysed seed populations. The resulting curvature parameters were subsequently used for quantitative comparisons among seed morphotypes and for the centroid-based selection of representative populations.

### 4.9. Statistical Analysis

#### 4.9.1. Morphometric Dataset and Hierarchical Structure

The morphometric dataset analysed in this study is provided in Supplementary Table S1 (Sheet 1). It comprises measurements from 2895 individual seeds belonging to 143 populations representing 85 Vitis vinifera cultivars. For each seed, the dataset includes the initial pedigree-based assignment to one of three reference groups (Hebén, Savagnin Blanc, or Muscat), cultivar name (VAR), population (POBL), sampling site (SITE), individual seed number (NUMBER), and the normalized Elliptic Fourier Descriptors corresponding to the first eight harmonics (A_1_–A_8_, B_1_–B_8_, C_1_–C_8_, and D_1_–D_8_).

To account for the hierarchical nesting of individual seeds within populations and cultivars, statistical analyses were structured to minimize pseudoreplication. Population-level means were used as the primary observational unit for multivariate comparisons among groups, while Linear Mixed-Effects Models (LMMs) were applied where appropriate, with population and/or cultivar included as random effects to account for non-independence among observations.

#### 4.9.2. *J* Index Classification

*J* index values were calculated automatically using the R workflow described in Section 4.7. The computational pipeline reconstructed each seed outline from its normalized Elliptic Fourier Descriptors and compared it with the three reference morphotypes (Hebén, Savagnin Blanc, and Muscat). During geometric alignment, the algorithm allowed minor adjustments in aspect ratio to optimize the correspondence between reconstructed seed outlines and reference models.

For each population, the mean *J* index ($\bar{J}$) was calculated for each of the three reference morphotypes. These three population-level values were then compared using a deterministic classification procedure based on their relative differences. A predefined critical difference margin (δ = 0.005) was used to determine whether the difference between the two highest *J* index values was sufficiently large to support an unambiguous assignment to a single reference morphotype.

Populations were assigned according to the following decision rules:

- Dominant groups (Groups I–III): a population was assigned to the reference morphotype with the highest *J* index when its value exceeded the second-highest *J* index by more than δ. Groups I, II, and III corresponded to the Hebén, Savagnin Blanc, and Muscat reference morphotypes, respectively.
- Co-dominant groups (Groups IVa and IVb): a population was classified as undifferentiated when the two highest *J* index values, not differing between them by more than δ, differed from the third value by more than δ, indicating comparable morphometric affinity to two of the three reference morphotypes.
- Undifferentiated morphotypes (Group V): a population was classified as undifferentiated when all three *J* index values differed by no more than δ, indicating comparable morphometric affinity to the three reference morphotypes.

Thus, the *J* index classification was based on the magnitude of differences among population-level similarity estimates rather than on an arbitrary assignment to the highest value alone. The procedure was designed to distinguish populations showing clear morphometric affinity to a single reference morphotype from those exhibiting overlapping or intermediate affinities.

#### 4.9.3. Selection of the Critical Difference Margin

The critical difference margin (δ = 0.005) was established as a predefined criterion based on the sampling precision of population-level *J* index estimates. Each population was represented by approximately 20 seeds, although the exact sample sizes varied and are reported in Supplementary Table S1. For each population, the *J* index was calculated as the mean of the individual seed-level *J* index values ($\bar{J}$). The standard error (SE) of the population mean was estimated as:

$$SE=\frac{s}{\sqrt{n}}$$

where s is the within-population standard deviation and n is the number of analyzed seeds. For the present dataset (n≈20), the within-population standard deviation typically ranged from 0.015 to 0.022, resulting in SE values of approximately 0.003–0.005.

These values indicate the expected magnitude of sampling uncertainty associated with population-level *J* index estimates. Accordingly, δ = 0.005 was adopted as a conservative resolution margin for interpreting small differences between competing reference morphotypes.

Differences between the two highest population-level *J* index values that were less than or equal to δ were therefore not considered sufficiently large to justify an unambiguous single-morphotype assignment. Instead, such populations were classified as co-dominant or undifferentiated according to the decision rules described in Section 4.9.2. This criterion provides a predefined and reproducible framework for distinguishing clear morphometric affinities from cases in which the observed differences are comparable to the expected sampling variation in the population means. The δ threshold was thus used as a classification-resolution criterion rather than as a formal statistical significance threshold. This distinction avoids interpreting the *J* index differences as the outcome of a conventional hypothesis test while providing an explicit quantitative basis for reproducible morphotype assignment.

#### 4.9.4. Selection of Representative Morphotypes

Following the *J* index classification, curvature values were calculated for all seeds belonging to Groups I, II, and III. Mean curvature values at the five homologous landmark positions were subsequently obtained for every population.

To identify representative morphotypes a multivariate centroid-based procedure was applied independently to each group. First, the centroid of each morphospace was calculated from the mean curvature values at the five landmark positions. Euclidean distances between each population and the corresponding group centroid were then computed in the five-dimensional curvature space as:

$$d_{i}=\sqrt{\sum_{k=1}^{5}{\left(x_{ik}-{\bar{x}}_{k}\right)}^{2}}$$

where $x_{ik}$ is the mean curvature value of population *i* at landmark *k*, and

$${\bar{x}}_{k}=\frac{\sum_{j=1}^{n_{I}}x_{jk}}{n_{I}}$$

the corresponding group centroid component, with $n_{I}$ the number of populations in the group (e.g., I).

The six populations exhibiting the shortest distances were selected as the representative morphotypes because they best reflected the central tendency of the corresponding group while minimizing the influence of atypical or extreme morphologies.

#### 4.9.5. Statistical Comparisons

Differences in curvature variables among morphotype groups were evaluated using the Kruskal–Wallis rank-sum test.

Whenever the overall test indicated significant differences (*p* < 0.05), pairwise comparisons were performed using Wilcoxon rank-sum tests (Mann–Whitney *U* tests). To control the family-wise error rate arising from multiple comparisons, *p*-values were adjusted using the Holm–Bonferroni sequential correction.

All statistical analyses, outline reconstructions, *J* index calculations, curvature analyses, clustering procedures, and graphical representations were performed in R [53].

## 5. Conclusions

This study presents a fully automated computational workflow implemented in R for high-throughput quantitative seed morphometrics using Elliptic Fourier Analysis (EFA). By integrating EFD-based outline reconstruction, automated *J* index comparison, continuous curvature profiling, and centroid-based selection of representative populations, the workflow provides a reproducible, quantitative framework for seed-shape characterization and morphotype identification while minimizing subjective visual classification. The approach is scalable to large germplasm collections and does not require manual placement of morphological landmarks.

Application of the workflow to 2895 seeds from 143 grapevine populations demonstrated that automated seed morphometrics captures biologically meaningful phenotypic variation associated with the major genomic haplotype groups considered in this study. The results also revealed populations showing intermediate or overlapping morphometric affinities, highlighting the continuous nature of seed-shape variation and the limitations of assigning complex phenotypes to discrete categories. Rather than replacing DNA-based approaches, automated seed morphometry provides an independent phenotypic framework that can complement genomic information in the characterization of grapevine diversity and the study of domestication.

Beyond its biological implications, the methodology provides a practical approach for large-scale phenotypic characterization of grapevine germplasm. The combination of standardized image acquisition and automated computational analysis can facilitate the screening of germplasm repositories, support cultivar characterization, and assist in the analysis of historical and archaeobotanical seed material. Because seed morphometry and genomic data provide complementary levels of biological information, their integration may improve the interpretation of relationships among genotype, phenotype, and environmental variation in grapevine.

More broadly, the computational strategy developed here is not restricted to *Vitis*. The combination of EFA-based outline reconstruction, automated *J* index calculation, curvature analysis, and centroid-based morphometric selection constitutes a general framework that can be adapted to other biological structures characterized by closed contours. Future studies integrating this approach with genomic, developmental, and environmental datasets will be important for testing the genetic basis of the observed morphological patterns and for clarifying the mechanisms underlying seed-shape variation. Such integration may contribute to the development of computational phenomics as a complementary approach to modern plant genomics.

## Figures and Tables

**Table 1:** Classification of grapevine seed populations before and after *J* index analysis. The upper section shows the initial assignment of the 143 populations based on published pedigree information and preliminary visual assessment of seed morphology. The lower section presents the revised classification obtained following automated *J* index analysis.

Step	Distribution of Populations
Initial classification	80 Hebén
	35 Savagnin Blanc
	28 Muscat
After *J* index analysis	41 Group I
	12 Group II
	21 Group III
	55 equally similar to Hebén and Savagnin Blanc (Group IVa)
	4 equally similar to Hebén and Muscat (Group IVb)
	10 equally similar to all three reference models (Group V)

**Table 2:** Populations assigned to Group I (Hebén) following *J* index-based classification. Group I comprises populations exhibiting the highest *J* index values relative to the Hebén reference morphotype. Population names include the cultivar designation followed, where applicable, by the collection site and/or year of sampling.

Alarije IMIDRA 2020	Muscat à Petits Grains Valbusenda
Aledo IMIDRA 2020	Planta Fina IMIDRA 2020
Blanquiliña IMIDRA 2020	Planta Fina IMIDRA 2024
Cadrete IMIDRA 2020	Quigat IMIDRA 2020
Esperó de Gall IMIDRA 2020	Quigat IMIDRA 2024
Forcallat IMIDRA 2020	Sauvignon Blanc Valbusenda
Hebén IMIDRA 2020	Sumol IMIDRA 2024
Hebén IMIDRA 2025	Tarragoní 2020
Italia IMIDRA 2020	Tempranillo Abadía Retuerta
Italia Moscatel Romano Valbusenda	Tempranillo Ayuso
Italia Valbusenda	Tempranillo IMIDRA 2020
Lariao INIAV Pt	Tempranillo INIAV Pt
Malvar IMIDRA 2020	Tinta de Toro Abadía Retuerta
Mantúo de pilas IMIDRA 2020	Torralba IMIDRA 2020
Merseguera IMIDRA 2020	Trepat IMIDRA 2024
Merseguera IMIDRA 2024	Verdejo Salamanca IMIDRA 2020
Miguel de Arco IMIDRA 2020	Verdejo Salamanca IMIDRA 2024
Miguel de Arco IMIDRA 2024	Verdil IMIDRA 2020
Miguel de Arco Valbusenda	Xarello IMIDRA 2020
Moscatel Angues IMIDRA 2020	Xarello IMIDRA 2024
Muscat à Petits Grains IMIDRA 2020	

**Table 3:** Populations assigned to Group II (Savagnin Blanc) following *J* index-based classification. Group II comprises populations exhibiting the highest *J* index values relative to the Savagnin Blanc reference morphotype. Population names include the cultivar designation followed, where applicable, by the collection site and/or year of sampling.

Albarín blanco IMIDRA 2020	Maturana Blanca IMIDRA 2020
Alfrocheiro IMIDRA 2020	Mollar Cano IMIDRA 2020
Alfrocheiro INIAV Pt	Muscat Hamburg IMIDRA 2020
Beba IMIDRA 2020	Muscat Hamburg IMIDRA 2025
Chenin Blanc Valbusenda	Prieto Picudo IMIDRA 2020
Kodrianka Valbusenda	Sabro 2020

**Table 4:** Populations assigned to Group III (Muscat) following *J* index-based classification. Group III comprises populations exhibiting the highest *J* index values relative to the Muscat reference morphotype. Population names include the cultivar designation followed, where applicable, by the collection site and/or year of sampling.

Airén IMIDRA 2020	Parduca IMIDRA 2020
Arinto INIAV Pt	Perrum INIAV Pt
Bobal IMIDRA 2020	Red Globe IMIDRA 2020
Borba IMIDRA 2020	Red Globe Valbusenda
Camarate Tinto INIAV Pt	Sauvignon Blanc Abadía Retuerta
Cariñena Mazuela IMIDRA 2020	Trincadeira das Pratas IMIDRA 2020
Chenin Blanc IMIDRA 2020	Verdejo IMIDRA 2020
Gouveio INIAV Pt	Verdelho INIAV Pt 50317
Matilde Valbusenda	Verdelho INIAV Pt 51509
Mazuela Valbusenda	Vijariego Blanco IMIDRA 2020
Muscat of Alexandria Valbusenda	

**Table 5:** Mean curvature values and Kruskal–Wallis test results for average curvature (AveCurv) and curvature at landmark positions P1–P5 in the six populations selected as representatives of Groups I, II, and III. Curvature values were calculated for all seeds from the six populations closest to the centroid of each morphometric group, and the table reports the corresponding mean values. Within each column, values followed by different letters differ significantly according to the Kruskal–Wallis test followed by pairwise Wilcoxon rank-sum tests with Holm–Bonferroni correction (*p* < 0.05).

	N	AveCurv	P1	P2	P3	P4	P5
Group I	140	1.46 ^c^	5.87 ^b^	−1.71 ^a^	1.33 ^c^	0.57 ^a^	3.14 ^b^
Group II	124	1.69 ^a^	6.41 ^a^	−2.80 ^c^	1.72 ^a^	0.58 ^a^	3.22 ^b^
Group III	117	1.63 ^b^	6.53 ^a^	−2.17 ^b^	1.44 ^b^	0.57 ^a^	3.47 ^a^

## Data Availability

The original contributions presented in this study are included in the article/Supplementary Materials.

## Supplementary Materials

Table S1 (Sheet 1) contains the group, cultivar, population, site, seed number and Fourier coefficients for the total 2895 seeds in this study. Table S1 (Sheet 2) contains the information related to cultivars and populations. Table S1 (Sheet 3) contains the results of the *J* index analysis with the adscription of populations to morphological groups. Six PDF files contain the outlines of all seeds grouped by populations, three of them correspond to the initial groups before *J* index analysis (Hebén, Savagnin Blanc and Muscat), and three to the groups formed after *J* index analysis (Groups I, II and III). Folders “Group I representative files”, “Group II representative files” and “Group III representative files” contain curvature data and images for representative seeds of Groups I, II and III. R Files Fouriercoefs.R, JIndex.R and Curvature.R contain respectively the scripts to obtain the Fourier coefficients from JPG images, to calculate the *J* index from Fourier coefficients, and to calculate curvature points from Fourier coefficients. R files classifiespopulations.R and sixmore representatives.R contain respectively the scripts to classify populations based on *J* index values and to select the six representative populations according to their Euclidean distance from the centroid of each morphotype. All these materials are available at https://zenodo.org/records/21626743 (accessed on 26 June 2026).
